# Evolutionarily Conserved and Divergent Roles of Unfolded Protein Response (UPR) in the Pathogenic *Cryptococcus* Species Complex

**DOI:** 10.1038/s41598-018-26405-5

**Published:** 2018-05-25

**Authors:** Kwang-Woo Jung, Kyung-Tae Lee, Anna F. Averette, Michael J. Hoy, Jeffrey Everitt, Joseph Heitman, Yong-Sun Bahn

**Affiliations:** 1 Department of Biotechnology, Yonsei University, Seoul, 03722 Republic of Korea; 2 Department of Molecular Genetics and Microbiology, Medicine, and Pharmacology and Cancer Biology, Duke University Medical Center, Durham, North Carolina 27710 USA; 3 Department of Pathology, Duke University Medical Center, Durham, North Carolina 27710 USA; 4 Present Address: Research Division for Biotechnology, Korea Atomic Energy Research Institute, Jeongeup, 56212 Korea

## Abstract

The unfolded protein response (UPR) pathway, consisting of the evolutionarily conserved Ire1 kinase/endonuclease and the bZIP transcription factor Hxl1, is critical for the pathogenicity of *Cryptococcus neoformans*; however, its role remains unknown in other pathogenic *Cryptococcus* species. Here, we investigated the role of the UPR pathway in *C. deuterogattii*, which causes pneumonia and systemic cryptococcosis, even in immunocompetent individuals. In response to ER stress, *C. deuterogattii* Ire1 triggers unconventional splicing of *HXL1* to induce the expression of UPR target genes such as *KAR2*, *DER1*, *ALG7*, and *ERG29*. Furthermore, *C. deuterogattii* Ire1 is required for growth at mammalian body temperature, similar to *C. neoformans* Ire1. However, deletion of *HXL1* does not significantly affect the growth of *C. deuterogattii* at 37 °C, which is in contrast to the indispensable role of *HXL1* in the growth of *C. neoformans* at 37 °C. Nevertheless, both *C. deuterogattii ire1*Δ and *hxl1*Δ mutants are avirulent in a murine model of systemic cryptococcosis, suggesting that a non-thermotolerance phenotypic trait also contributes to the role of the UPR pathway in the virulence of pathogenic *Cryptococcus* species. In conclusion, the UPR pathway plays redundant and distinct roles in the virulence of members of the pathogenic *Cryptococcus* species complex.

## Introduction

The pathogenic *Cryptococcus* species complex consists of two major lineages: *Cryptococcus neoformans* and *Cryptococcus gattii*^1^. *C. neoformans* causes life-threatening meningoencephalitis, mainly in the immunocompromised population, whereas *C. gattii* causes diseases in both immunocompetent and immunocompromised individuals, as shown by the outbreak in British Columbia^2^. The *C. neoformans* lineage is now speciated into *C. neoformans* (molecular type VNI/AFLP1, VNII/AFLP1A/AFP1B, and VNB/AFLP1; serotype A; formerly *C. neoformans* var. *grubii*), *C. deneoformans* (VNIV/AFLP2; serotype D; formerly *C. neoformans* var. *neoformans*), and *C. deneoformans* and *C. neoformans* hybrids (VNIII/AFLP3, serotype AD hybrid)^3–7^. *C. neoformans* strains are generally more virulent and more frequently found worldwide compared with *C. deneoformans* strains^1^. The *C. gattii* lineage is currently delineated as five species, *C. gattii* (VGI/AFLP4; serotype B), *C. deuterogattii* (VGII/AFLP6; serotype B), *C. bacillisporus* (VGIII/AFLP5; serotype B and C), *C. tetragattii* (VGIV/AFLP7; serotype C), and *C. decagattii* (VGIV/AFLP10; serotype C)^3–7^. *C. gattii* including strain WM276 is a species commonly isolated from natural environments and patients; *C. deuterogattii* including strain R265 is predominant in the Vancouver outbreak^8,9^.

The *C. gattii* lineage exhibits distinct biochemical, ecological, and pathological features compared to the *C. neoformans* lineage. The two lineages have different capabilities to utilize nitrogen and carbon sources. Although creatinine is used as the source of nitrogen in both species, creatinine deiminase, which is required for creatinine metabolism, is regulated differently. Its expression is suppressed by ammonia in *C. neoformans*, but not in *C. gattii*^10^. Glycine is utilized as both a carbon and nitrogen source in *C. gattii*, but only as a nitrogen source in *C. neoformans*^11^. Given that most *C. gattii* strains, but none of *C. neoformans*, are resistant to L-canavanine, CGB (canavanine-glycine-bromothymol blue) medium is widely used to distinguish the two species^12^. Pigeon guano, a well-known ecological niche of *C. neoformans*, supports the growth of both species. On solid pigeon guano-containing medium, both *C. neoformans* and *C. gattii* produce melanin; however, spore production is more robust in *C. neoformans* than in *C. gattii*^13^. The mating efficiency of *C. gattii* is increased on media containing plant-derived substrates such as those of eucalyptus and *Arabidopsis*^14^. *C. neoformans* primarily causes meningitis through central nervous system infection following pulmonary infection, and *C. gattii* commonly causes pulmonary infection^15,16^. During murine infection, the primary target organ of *C. neoformans* is different from that of *C. gattii*^17^: After infection through pulmonary route, *C. gattii* grows faster in the lung and causes pneumonia, while *C. neoformans* moves faster and efficiently to the brain and causes meningoencephalitis^17^.

In spite of such pathobiological differences between *C. neoformans* and *C. gattii* species complexes, signalling pathways governing their virulence have been more extensively studied in *C. neoformans* than in *C. gattii*, partly because the *C. neoformans* H99 strain has been more widely used as a reference strain for pathogenic *Cryptococcus* species. Nevertheless, several evolutionarily conserved signalling pathways have been shown to be critical for the pathobiology of both species^2^. For example, the cAMP/PKA pathway is involved in the production of two major virulence factors: capsule and melanin^18–20^. The calmodulin/calcineurin pathway plays conserved roles in regulating thermotolerance, virulence, and cell wall/membrane integrity in both species^21–23^.

We recently reported that the unfolded protein response (UPR) pathway is one of the critical virulence-regulating signalling pathways in *C. neoformans*^24^. In *C. neoformans*, it consists of the evolutionarily conserved Ire1 kinase/endonuclease and its downstream bZIP transcription factor Hxl1. Notably, Hxl1 is structurally and functionally divergent from other previously reported Ire1 downstream transcription factors, Hac1 in *Saccharomyces cerevisiae* and Xbp1 in humans. Upon ER stress, the activated Ire1 eliminates the intron of the unspliced *HXL1* mRNA (*HXL1*^u^) in a splicesome-independent manner, thereby increasing spliced *HXL1* mRNA (*HXL1*^s^) transcripts that can be translated to the active Hxl1 transcription factor required for induction of UPR target genes. Therefore, perturbation of the UPR pathway, e.g. using tunicamycin (TM) and dithiothreitol (DTT) treatment, renders *C. neoformans* extremely susceptible to ER stress and cell wall-destabilizing agents, e.g. to calcofluor white (CFW) and Congo red (CR)^24^. Most notably, both *ire1*∆ and *hxl1*∆ mutants are unable to grow at the mammalian body temperature of 37 °C and are thereby completely avirulent in a murine model of systemic cryptococcosis^24^, suggesting that the UPR pathway, particularly Hxl1, could be a promising antifungal drug target. We also reported that the UPR pathway regulates Kar2, an ER-resident molecular chaperone, to counteract ER and cell wall stress and control thermotolerance and azole drug resistance^25^. Although Hxl1 is a *bona fide* transcription factor downstream of Ire1, Ire1 plays both Hxl1-dependent and -independent functions in modulating stress responses, virulence, and differentiation^24,26^. For instance, Ire1, but not Hxl1, regulates opposite- and unisexual mating and capsule production in a Rim101-dependent manner in *C. neoformans*^26^.

Despite the critical role of the UPR pathway in the virulence of the *C. neoformans* lineage, it remains unknown whether the function of the UPR pathway is conserved in the *C. gattii* lineage, how it is regulated, and whether it is critical for the virulence of the other pathogenic *Cryptococcus* species. To address these questions, we have identified *IRE1* and *HXL1* genes in *C. deuterogattii* and characterized their *in vitro* and *in vivo* roles. Here, we demonstrate that the UPR pathway plays evolutionarily conserved but different roles in *C. deuterogattii* and *C. neoformans*; however, it is still absolutely necessary in the virulence of *C. deuterogattii*.

## Results

### Identification of the Ire1 ortholog in *C. deuterogattii*

**Figure 1:**
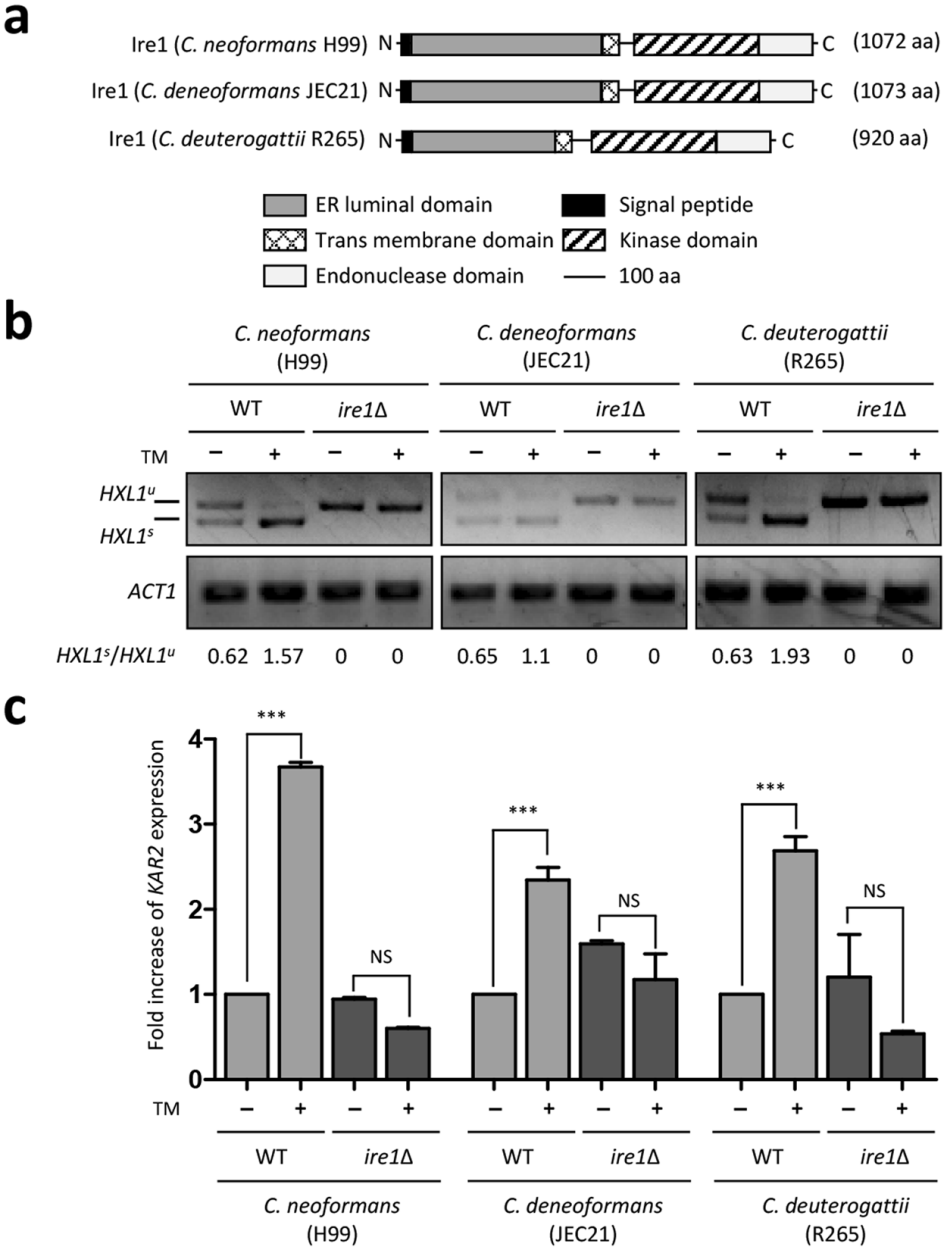
The evolutionarily conserved UPR pathway in the pathogenic *Cryptococcus* species complex. (**a**) The conserved domain structure of the *C. neoformans* H99 strain, *C. deneoformans* JEC21 strain, and *C. deuterogattii* R265 strain Ire1. (**b**) RT-PCR analysis of *HXL1* splicing in the pathogenic *Cryptococcus* species complex during ER stress. The cDNA was synthesized from total RNA from *Cryptococcus* cells treated with or without TM (0.3 μg/mL) for 1 h as the template. The grouping of gels is cropped from different parts of the same gel and the original images are shown in the Supplementary Fig. 1. (**c**) Quantitative RT-PCR analysis of *KAR2* in the pathogenic *Cryptococcus* species complex during ER stress. The qRT-PCR was performed with cDNA from wild-type strains and *ire1*Δ mutants treated or not treated with TM (0.3 μg/mL) for 1 h. Duplicate technical experiments with two independent biological samples were performed. Representative images from independent experiments for *KAR2* gene are shown. Error bars indicate standard deviations. Asterisks indicate statistical significance of differences in expression levels of each gene (***P < 0.001; NS, not significant).

To identify the Ire1 ortholog in the *C. deuterogattii* strain R265, we performed BLASTp searches using serotype A Ire1 as the query in the *C. gattii* genome database (https://www.broadinstitute.org/scientific-community/science/projects/fungal-genome-initiative/cryptococcus-gattii-genome-project-0). *C. deuterogattii* R265 contains CNBG_0073.2, which is orthologous to *C. neoformans* Ire1 (score 1571.6, e-value: 0). Notably, the *C. deuterogattii* R265 Ire1 ortholog is 152 and 153 amino acids shorter than the *C. neoformans* strain H99 and *C. deneoformans* JEC21 Ire1 orthologs, respectively (Fig. 1a). Based on protein domain analysis by Pfam (http://pfam.sanger.ac.uk/), the *C. deuterogattii* Ire1 ortholog is predicted to contain kinase and endonuclease domains like the *C. neoformans* lre1 ortholog (Fig. 1a).

In our previous study, we identified the Hxl1 ortholog in *C. deuterogattii* R265 (CNBG_4842.2) and confirmed that ER stress-induced unconventional splicing of *HXL1* mRNA occurs^24^. To test whether the unconventional splicing of *HXL1* mRNA is mediated by Ire1 in *C. deuterogattii*, we constructed the *ire1*∆ mutant of the R265 strain and then monitored *HXL1* splicing in response to the ER stress-inducing agent tunicamycin (TM). Similar to *C. neoformans*, *C. deneoformans* and *C. deuterogattii* strains exhibited an Ire1-dependent *HXL1* splicing event during ER stress (Fig. 1b and Supplementary Fig. 1). During ER stress, the molecular chaperone *KAR2* is induced in the Ire1-Hxl1-dependent UPR pathway in *C. neoformans*^24^. To confirm that *KAR2* induction is regulated by Ire1, we monitored the expression level of *KAR2* in both R265 strain and the *ire1*∆ mutant when cells were exposed to TM. In accordance with the *KAR2* induction in *C. neoformans*, expression of *KAR2* was upregulated in *C. deuterogattii*, but not in the *ire1*∆ mutant (Fig. 1c). These data indicate that Ire1-mediated UPR induction is widely conserved in the pathogenic *Cryptococcus* species complex.

### Ire1 and Hxl1 play evolutionarily conserved roles in ER stress response and adaptation

**Figure 2:**
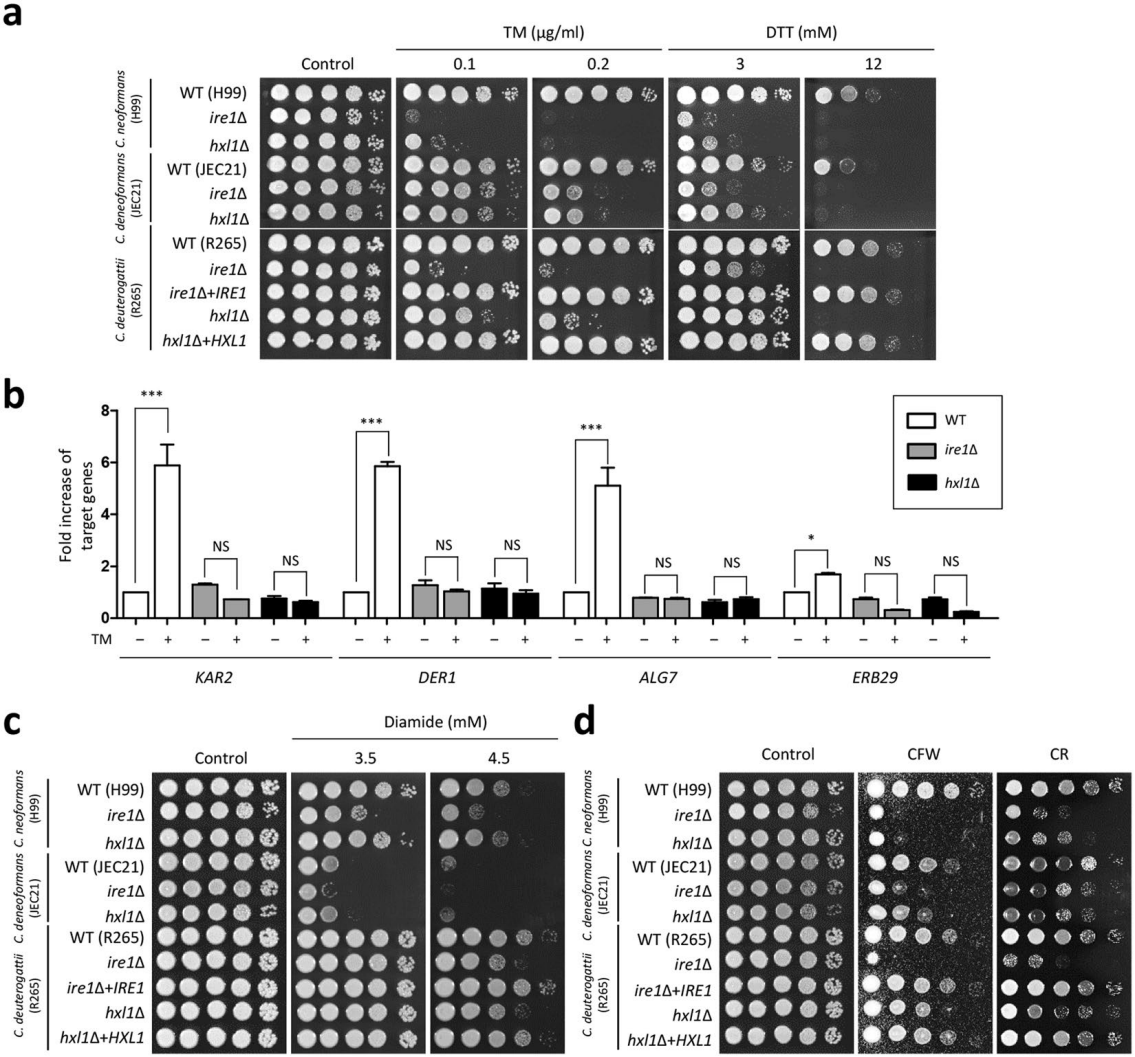
The evolutionarily conserved functions of the pathogenic *Cryptococcus* UPR pathway in response to ER stress, cell wall stress, and diamide resistance. (**a**,**c** and **d**) *Cryptococcus* cells were cultured overnight at 30 °C and washed with sterilized water. Cells were serially diluted (dilutions from 1 to 10^−4^) and then spotted onto YPD medium containing the indicated concentrations of ER stress inducers (TM or DTT), cell wall destabilizers (CFW or CR), or diamide. The plates were incubated at 30 °C and photographed daily for 2–4 days. The two images split by a horizontal white line in each spot assay were obtained from the same plate. (**b**) *C. deuterogattii* strains were grown in liquid YPD medium to mid-log phase (OD_600_ = 0.6), exposed to TM (0.3 μg/mL) for 1 h, and harvested. The cDNA was synthesized from total RNA in cells treated or not treated with TM. Duplicate technical experiments with two biological samples were performed. Representative images from independent experiments for the *KAR2, DER1, ALG7*, and *ERV29* genes are shown. Error bars indicate standard deviations. Asterisks indicate the statistical significance of differences in expression levels of each gene. (***P < 0.001; *P < 0.05; NS, not significant) Strain information: WT (H99, JEC21, and R265), *ire1*Δ mutant (YSB552, YSB2886, and YSB1889), *hxl1*Δ mutant (YSB723, YSB2030, and YSB1891), *ire1*Δ + *IRE1* complemented strains (YSB3158), and *hxl1*Δ + *HXL1* complemented strains (YSB2957).

To address whether UPR activation is required for ER stress response and adaptation in *C. deuterogattii*, we disrupted the *HXL1* gene in the R265 strain. Expectedly, *C. deuterogattii ire1*Δ and *hxl1*Δ mutants displayed severe growth defects in response to ER stress inducers such as TM and DTT, whereas their complemented strains (*ire1*Δ + *IRE1* and *hxl1*Δ + *HXL1*) exhibited levels of ER stress resistance restored to the wild-type level (Fig. 2a). Similar to the phenomenon observed in *C. neoformans* UPR mutants, the *ire1*Δ mutant was more sensitive to ER stress than the *hxl1*Δ mutant of the R265 strain, indicating that Ire1 plays a role in modulating ER stress in both Hxl1-dependent and -independent manners in *C. deuterogattii*. ER stress causes induction of *KAR2* expression through Ire1-mediated *HXL1* splicing in the pathogenic *Cryptococcus* species complex (Fig. 1b and c). Supporting the finding that the UPR pathway is required for counteracting ER stress in *C. deuterogattii*, deletion of *IRE1* or *HXL1* abolished the induction of *KAR2* in response to ER stress in the R265 strain (Fig. 2b).

Our previous study reported that some *C. neoformans* UPR-target genes, including *KAR2*, harbor the UPR element (UPRE) and UPRE-like sequence in their promoter and their expression is regulated in response to ER stress and temperature upshift^24^. These include genes involved in protein secretion and lipid linked *N*- and *O*-glycosylation^24^. Therefore, we examined whether the other known *C. neoformans* UPR target genes are also regulated by the *C. deuterogattii* UPR pathway. First, we confirmed the presence of UPRE and UPRE-like sequences in the promoters of each putative UPR target (Supplementary Fig. S2). Similar to *C. neoformans*, expression levels of *DER1* (ER-associated degradation), *ALG7* (lipid-linked *N*-oligosaccharyltransferase), and *ERV29* (ER to Golgi vesicle-mediated transport) were significantly increased in the R265 strain, but not in the *ire1*Δ or *hxl1*Δ mutants (Fig. 2b), suggesting that that they are ER stress-dependent UPR target genes in *C. deuterogattii*. However, expression changes of other putative UPR downstream genes such as *PPS1*, *SOD2*, *PMT1*, *PMT4*, *CHS2*, *SEC61*, *OST1*, and *WBP1* were not significantly different between wild- type and UPR mutants in both *C. neoformans*^24^ and *C. deuterogattii* (Supplementary Fig. S3).

Our previous study revealed that Ire1 has an Hxl1-independent function in modulating resistance to diamide, which is a thiol-specific oxidant inducing unnatural disulfide formation in a protein or between proteins and triggering protein misfolding, in *C. neoformans*^24^. To examine whether this phenomenon is also evolutionarily conserved, we monitored growth of UPR mutants under diamide treatment in other pathogenic *Cryptococcus* species. Similar to the case in *C. neoformans*, the *C. deneoformans ire1*Δ mutant, but not *hxl1*Δ mutant, was more susceptible to diamide than wild-type strain (Fig. 2c). In *C. deuterogattii*, however, the *ire1*∆ mutant was as sensitive to diamide as the *hxl1*∆ mutants, suggesting that Ire1 controls diamide sensitivity in an Hxl1-dependent manner in *C. deuterogattii*. Another notable phenotype observed in the UPR mutants is the increased susceptibility to cell wall-damaging agents^24^. *C. deneoformans ire1*Δ and *hxl1*Δ mutants showed increased susceptibility, albeit to a lesser extent compared to *C. neoformans ire1*Δ and *hxl1*Δ mutants, to CFW and CR (Fig. 2d). *C. deuterogattii ire1*Δ and *hxl1*Δ mutants also exhibited increased susceptibility to CFW and CR (Fig. 2d). Notably, however, *C. deuterogattii ire1*Δ mutants displayed higher susceptibility to CFW and CR than the *hxl1*Δ mutants (Fig. 2d). In fact, the *C. deuterogattii hxl1*Δ mutants showed only a weakly increased susceptibility to CFW and CR (Fig. 2d). Taken together, all these data suggest that the regulatory mechanism of the *C. deuterogattii* UPR pathway for ER stress response and adaptation is generally conserved, albeit with some modification, compared to the *C. neoformans* UPR pathway.

### Ire1, but not Hxl1, is critical for the survival of *Cryptococcus deuterogattii* at mammalian body temperature

**Figure 3:**
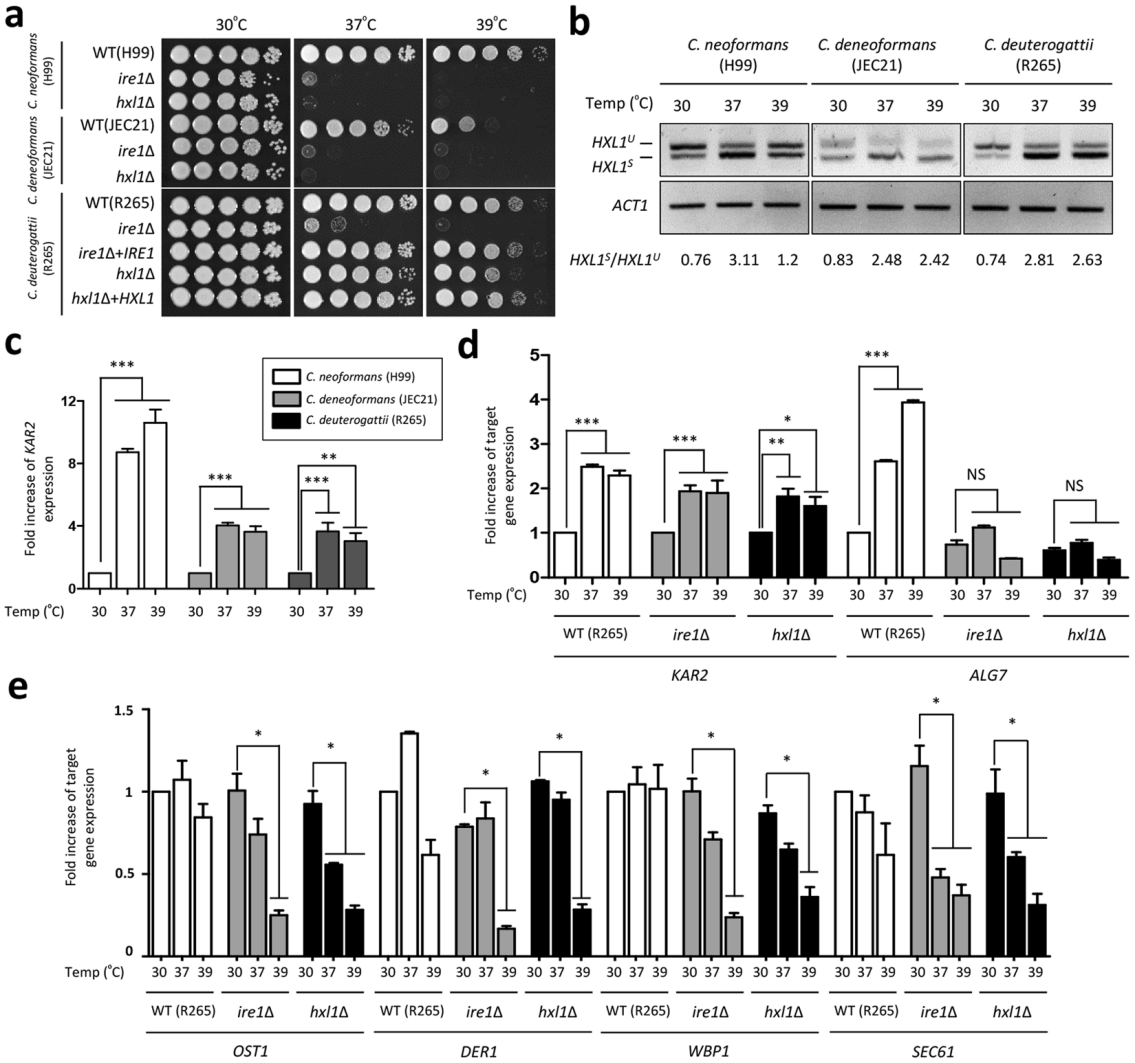
The conserved and distinct functions of the UPR pathway in the thermotolerance of the pathogenic *Cryptococcus* species complex. (**a**) For the thermotolerance test, cells were cultured in YPD liquid medium at 30 °C for 16 h. Each cell was serially diluted (dilutions from 1 to 10^−4^) and spotted onto YPD medium. Strains were further incubated at 30 °C, 37 °C, and 39 °C. The two images split by a horizontal white line in each spot assay were obtained from the same plate. (**b**) RT-PCR analysis of *HXL1* splicing in high temperature conditions. The cDNA was synthesized from the total RNA extracted from cells exposed to temperature upshift for 1 h. The specific primers for the amplification of *HXL1* and *ACT1* are described in Supplementary Table 3. The grouping of gels is cropped from different parts of the same gel and the original images are shown in the Supplementary Fig. 4. (**c**) *KAR2* was induced under temperature upshift. The qRT-PCR was performed with cDNA from wild-type strains upon temperature upshift (from 30 °C to 37 °C and from 30 °C to 39 °C) for 1 h. (**d** and **e**) Quantitative reverse transcription-PCR (qRT-PCR) analysis of putative UPR genes in *C. deuterogattii* during thermal shock. The cDNA was synthesized with total RNA from the wild-type R265 strain and *ire1*Δ and *hxl1*Δ mutants upon temperature upshift (from 30 °C to 37 °C and from 30 °C to 39 °C). Duplicate technical experiments with two or more independent biological samples were performed. Representative data from independent experiments for UPR putative gene are presented. Error bars indicate standard deviations. Asterisks indicate statistical significance of differences in expression levels of each gene (*P < 0.05; **P < 0.01; ***P < 0.001).

In our previous study, *C. neoformans* and *C. deneoformans ire1*Δ and *hxl1*Δ mutants exhibited growth defects at 37 °C: these mutations are believed to be the most critical factors accounting for their avirulence^24,26^. Similarly, the *C. deuterogattii ire1*Δ mutant exhibited severe growth defects at high temperature (37 °C and 39 °C), compared to the wild-type and its complemented strains (Fig. 3a), indicating that the role of Ire1 in thermotolerance is evolutionarily conserved in the pathogenic *Cryptococcus* species complex. Strikingly, however, the *C. deuterogattii hxl1*Δ mutant showed almost wild-type levels of thermotolerance at 37 °C (Fig. 3a). At higher temperature (39 °C), the *C. deuterogattii hxl1*Δ mutant showed subtly increased sensitivity. This is in stark contrast to *C. neoformans* and *C. deneoformans hxl1*Δ mutants that exhibited even higher thermosensitivity than the *ire1*Δ mutants (Fig. 3a). This phenomenon suggests that *C. deuterogattii* R265 Ire1 regulates thermotolerance through downstream transcription factors other than Hxl1.

Given that *C. deuterogattii* Hxl1 has a minor role in thermosensitivity, we wondered whether an *HXL1* splicing event occurs in *C. deuterogattii* during temperature upshift. In *C. neoformans*, temperature upshift induced splicing of *HXL1* mRNA (Fig. 3b and Supplementary Fig. 4). Compared to the splicing event of *HXL1* mRNA during ER stress, unspliced *HXL1* mRNA was still observed at 37 °C and 39 °C, although spliced *HXL1* mRNA was dominant. In *C. deneoformans*, however, spliced *HXL1* mRNA was significantly increased upon temperature upshift. Similar to *C. deneoformans*, the spliced *HXL1* mRNA was dominant at 37 °C and 39 °C in *C. deuterogattii* (Fig. 3b). Previous studies reported that the expression level of *KAR2* is up-regulated during thermal shock^24^. The constitutive *KAR2* overexpression partially rescued thermotolerance in *C. neoformans ire1*Δ and *hxl1*Δ mutants^25^. In line with the previous results, *KAR2* was induced upon temperature upshift in *C. deuterogattii* (Fig. 3c). To demonstrate whether the difference in thermosensitivity between *C. deuterogattii ire1*Δ and *hxl1*Δ mutants was attributable to the level of *KAR2* expression, we monitored its expression levels during the temperature upshift. However, the expression patterns of *KAR2* appeared to be indistinguishable between R265 strain and UPR mutants (Fig. 3d), suggesting that *KAR2* induction by thermal stress is independent of the UPR pathway in *C. deuterogattii* and not responsible for thermosensitivity of the *ire1*Δ mutant.

We monitored expression levels of other putative UPR target genes upon temperature upshift in *C. deuterogattii*. In contrast to *KAR2*, *ALG7* expression was upregulated upon temperature upshift in Ire1- and Hxl1-dependent manners (Fig. 3d). The expression of *OST1* (lipid-linked *N*-oligosaccharyltransferase)*, DER1* and *WBP1* (lipid-linked *N*-oligosaccharyltransferase) were decreased in both *ire1*∆ and *hxl1*∆ mutants at 39 °C (Fig. 3e). In the case of *OST1*, its expression was also decreased in the *hxl1*∆ mutants at 37 °C. Notably, expression of *SEC. 61* (translocation of misfolded proteins out of ER) was decreased in both *ire1*∆ and *hxl1*∆ mutants at 37 °C and 39 °C (Fig. 3e). The expression patterns of other putative UPR downstream genes including *PPS1*, *SOD2*, *PMT1*, *ERV29*, *CHS2*, and *PMT4* in the R265 strain were similar to those in both *ire1*∆ and *hxl1*∆ mutants upon temperature upshift (Supplementary Fig. 5). Based on the fact that the *C. deuterogattii hxl1*Δ mutant exhibited only a minor growth defect at high temperature, Alg7, Ost1, Der1, Wbp1, and Sec61 play a minor role in thermotolerance and other UPR target(s) may have a major role in the UPR-dependent-thermotolerance of *C. deuterogattii*.

### Evolutionarily conserved and divergent roles of Ire1 and Hxl1 in antifungal drug resistance

**Figure 4:**
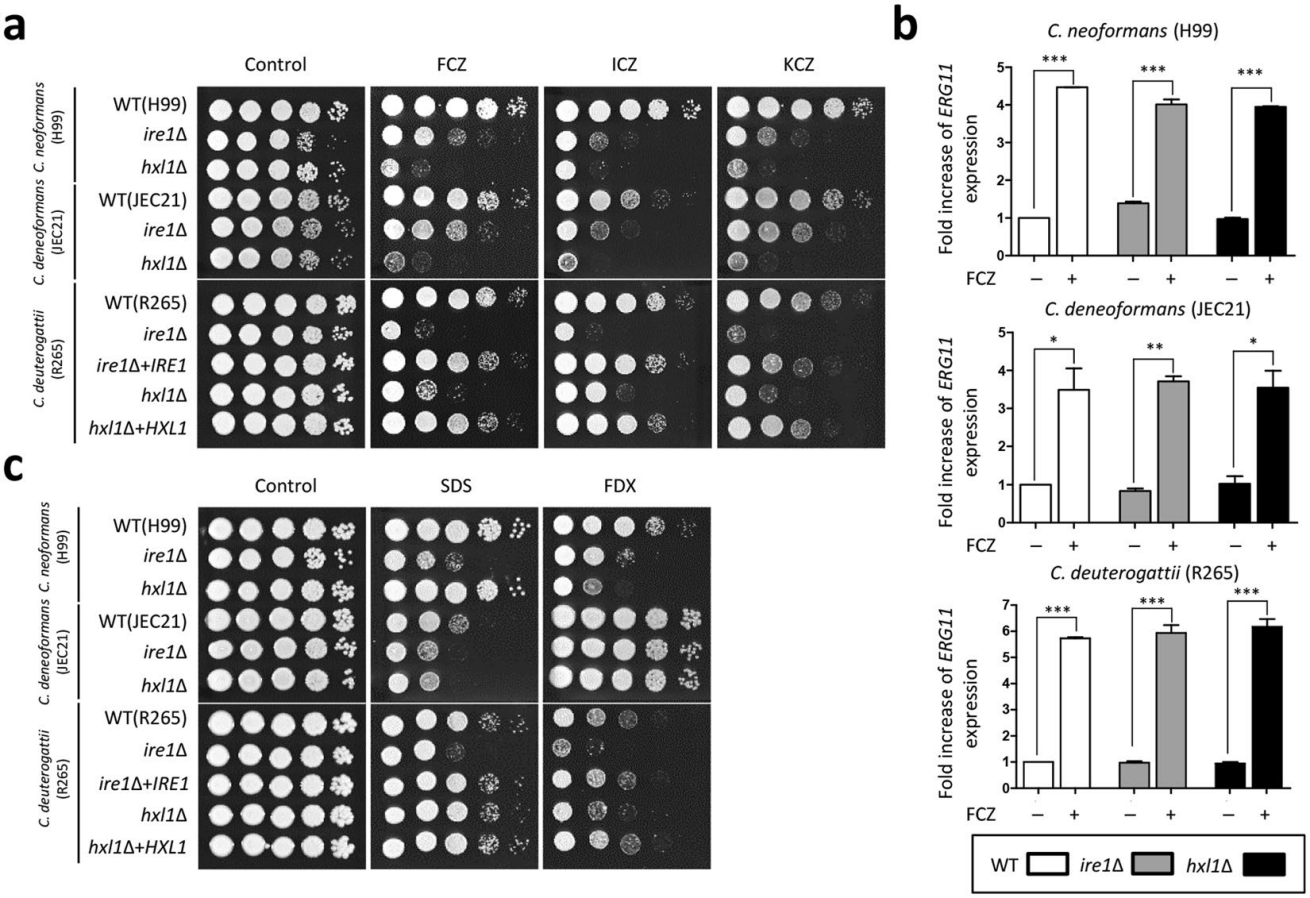
The pathogenic *Cryptococcus* UPR pathway plays a role in antifungal drug resistance, affecting membrane stability. (**a** and **c**) Strains were incubated in liquid YPD medium at 30 °C overnight. Cells were serially diluted and spotted onto YPD medium containing the indicated concentrations of azole drugs (FCZ (fluconazole, 5 μg/mL), ICZ (itraconazole, 0.025 μg/mL), and KCZ (ketoconazole, 0.1 μg/mL)) or cell membrane destabilizers (FDX (1 μg/mL) and SDS (0.03%)). Plates were further incubated at 30 °C for 2–4 days and photographed daily. The two images split by a horizontal white line in each spot assay were obtained from the same plate. (**b**) The qRT-PCR analysis for measuring *ERG11* expressions in wild-type and *ire1*Δ and *hxl1*Δ mutants of the pathogenic *Cryptococcus* species complex. *ERG11* induction is not required for the UPR pathway in the pathogenic *Cryptococcus* species complex. The cDNA was synthesized from total RNA in cells treated or not treated with fluconazole (10 μg/mL). Duplicate technical experiments with two biological samples were performed. Representative images from independent experiments for *ERG11* gene are shown. Error bars indicate standard deviations. Asterisks indicate the statistical significance of differences in expression levels of *ERG11* (*P < 0.05; **P < 0.01; ***P < 0.001).

Our previous studies showed that *C. neoformans ire1*Δ and *hxl1*Δ mutants exhibit increased susceptibility to azole drugs^24^. Their azole susceptibility is partially suppressed when *KAR2* is constitutively overexpressed^25^. Similarly, the *C. deuterogattii ire1*Δ and *hxl1*Δ mutants displayed growth defects in response to fluconazole, itraconazole, and ketoconazole (Fig. 4a). Notably, the deletion of *IRE1* made the R265 strain more susceptible to azoles than that of *HXL1*, which was in contrast to the finding that the *hxl1*Δ mutants were more susceptible to azoles than the *ire1*Δ mutants in *C. neoformans* and *C. deneoformans* (Fig. 4a). This indicates that the UPR components play conserved but different roles in azole drug resistance among the pathogenic *Cryptococcus* species complex.

Fluconazole treatment induces expression of ergosterol biosynthesis genes to counteract the inhibition of ergosterol biosynthesis in *C. neoformans*^27^. To determine whether the UPR pathway affects azole drug resistance by controlling the induction of *ERG* genes, we measured the expression levels of *ERG* genes in the pathogenic *Cryptococcus* UPR mutants after fluconazole treatment. We found that *ERG3* and *ERG11* were normally induced by fluconazole treatment in UPR mutants (Fig. 4b and Supplementary Fig. 6), indicating that the UPR pathway is not likely to be directly involved in ergosterol biosynthesis. As Erg11 inhibition by azoles allow toxic sterol intermediates to be incorporated into the cell membrane and then prevents cell proliferation^28^, we hypothesized that the UPR pathway could be involved in azole drug resistance by regulating cell membrane stability. Similar to the *C. neoformans ire1*Δ mutant, the *C. deuterogattii ire1*Δ mutant exhibited growth retardation in the presence of SDS, an ionic detergent that disrupts cell membrane integrity (Fig. 4c). In contrast, the *C. deuterogattii hxl1*Δ mutant exhibited wild-type levels of SDS resistance (Fig. 4c). Fludioxonil, which induces hyperactivation of the HOG pathway and thereby increases intracellular turgor pressure and affects cell membrane stability, inhibits the growth of *C. neoformans*, whereas it does not affect *C. deneoformans*^29^. *C. deuterogattii* UPR mutants were also susceptible to fludioxonil like *C. neoformans* UPR mutants (Fig. 4c). Notably, the *ire1*Δ mutant was more susceptible to fludioxonil than the *hxl1*Δ mutant in *C. deuterogattii*, which is in contrast to the finding that the *hxl1*Δ mutant was more susceptible to fludioxonil than the *ire1*Δ mutant in *C. neoformans* (Fig. 4c). Therefore, the results indicate that the role of the UPR pathway in cell membrane stability could contribute to azole resistance of the pathogenic *Cryptococcus* species. However, other factors may also indirectly contribute to the role of the UPR pathway in azole resistance.

### The *Cryptococcus* UPR pathway plays a minor role in capsule and melanin production

**Figure 5:**
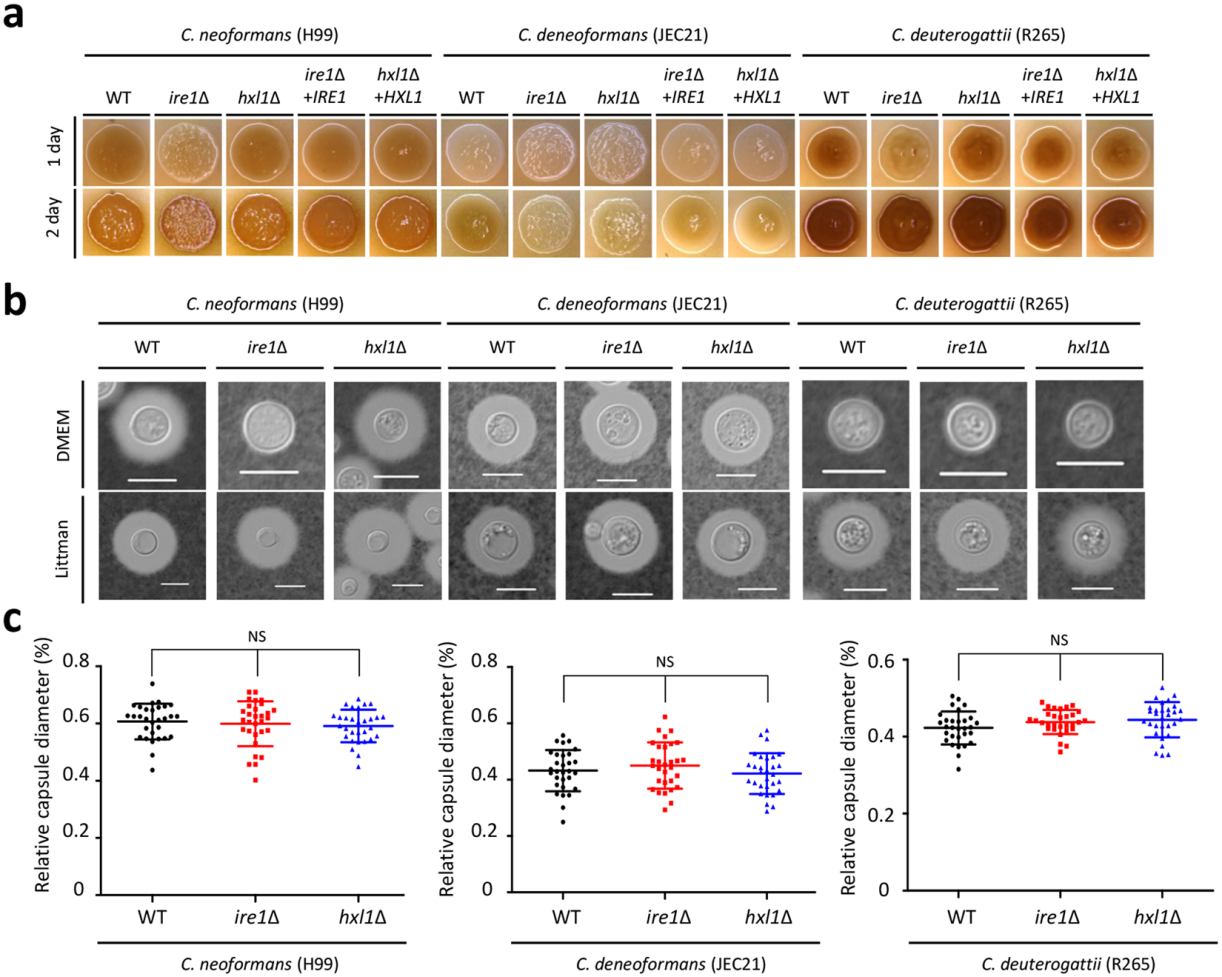
The UPR pathway affects capsule and melanin production in a species-dependent manner. (**a**) Each strain was cultured at 30 °C overnight and spotted onto solid Niger seed agar medium containing the indicated concentration of glucose (0.1%). Cells were photographed with a digital camera for 1–2 days. (**b**) Cells were incubated in solid-agar based DME medium (upper panel) or liquid Littman medium (lower panel) at 30 °C for 2 days. After incubation, capsules were stained with India ink and visualized using an Olympus BX51 microscope equipped with a SPOT insight digital camera. The white bar indicates 10 μm. (**c**) Quantitative measurement of the relative capsule diameter in the UPR mutants. The scatter plot indicates relative capsule diameter of strains incubated in liquid Littman medium. A total of 30 cells in each strain were measured for capsule production. *P*-values were determined by one-way analysis of variance with Bonferroni’s multiple-comparison test. Error bars indicate standard deviations. NS, not significant.

The pathogenic *Cryptococcus* species complex has several virulence factors responsible for survival and proliferation within the host^30^. Among these, melanin has antiphagocytic and antioxidant activities and the polysaccharide capsule protects the cells from being phagocytosed by host phagocytic cells^31,32^. To address the role of the UPR pathway in the formation of virulence factors, we examined the capability of the UPR mutants to produce melanin and capsules. We found that the *C. neoformans ire1*Δ mutant, but not the *hxl1*Δ mutant, produced melanin in a delayed manner, but eventually produced wild-type levels of melanin (Fig. 5a). Similarly, the *C. deuterogattii ire1*Δ mutant, but not the *hxl1*Δ mutant, showed delayed melanin synthesis (Fig. 5a). In *C. deneoformans*, however, both *ire1*Δ and *hxl1*Δ mutants exhibited decreased levels of melanin production (Fig. 5a).

We previously reported that the *C. neoformans ire1*Δ mutant, but not the *hxl1*Δ mutant, displayed capsule defects in Dulbecco’s modified Eagle’s (DME) medium^24^. In contrast, both *C. deneoformans* and *C. deuterogattii* UPR mutants produced wild-type levels of capsules in DME medium (Fig. 5b). As capsule production levels in *C. deuterogattii* were not evident in DME medium, we used Littman medium, which is known to induce capsule formation much more strongly than DME medium^33^. *C. deuterogattii* UPR mutants incubated in Littman medium exhibited wild-type levels of capsule (Fig. 5b). Interestingly, as opposed to the reduced capsule production of *C. neoformans ire1*Δ mutant in DME medium, wild-type levels of capsules were observed in the *ire1*Δ mutants in Littman medium (Fig. 5b). Quantitative measurements of relative capsule size from cells cultured in Littman medium also confirmed that all UPR mutants produced wild-type levels of capsule (Fig. 5c). Thus, the UPR pathway appears to play a minor role in melanin and capsule production in the pathogenic *Cryptococcus* species complex.

### Evolutionarily conserved role of the UPR pathway in *Cryptococcus* species complex virulence

**Figure 6:**
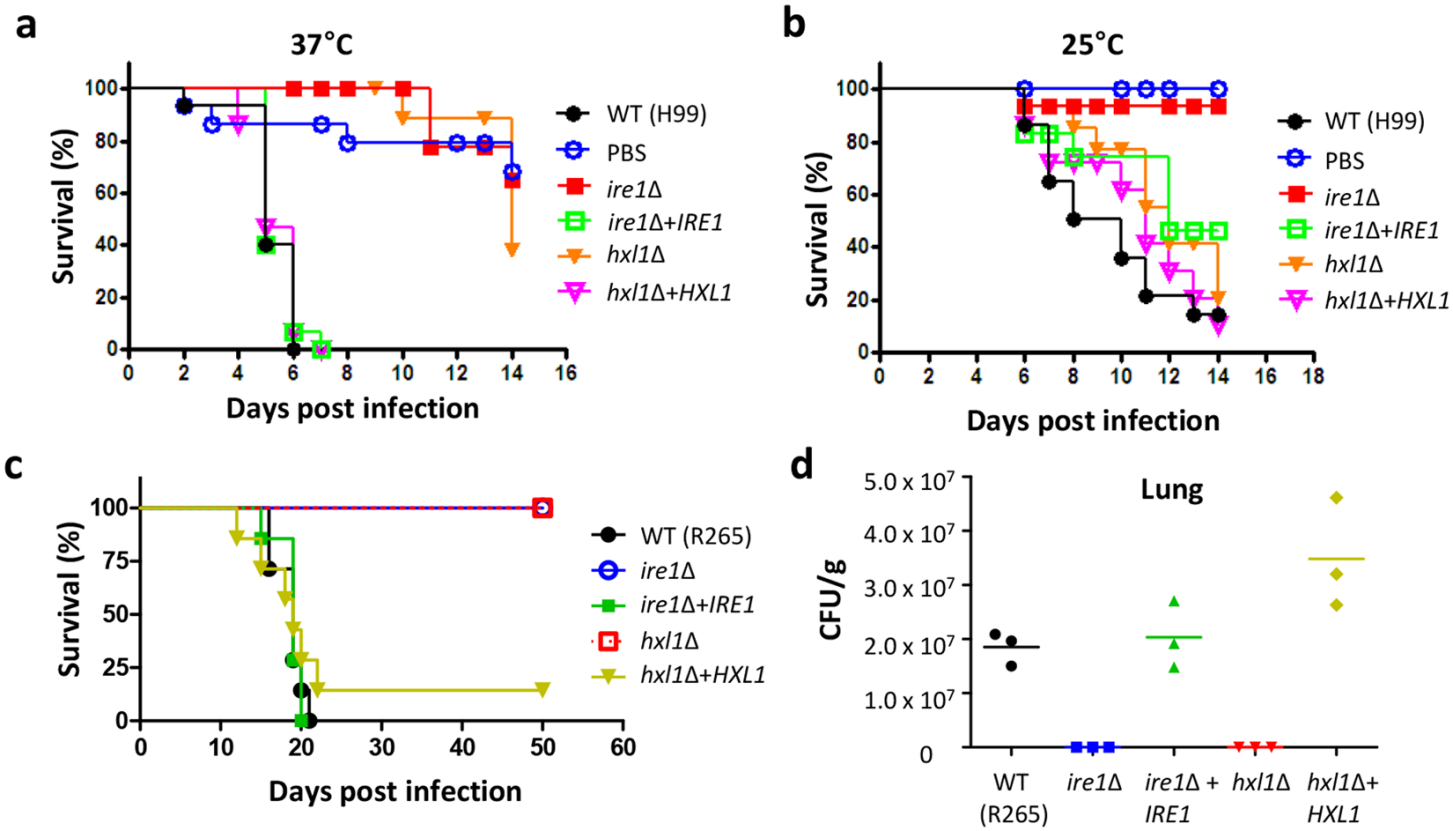
The UPR pathways governs the virulence of the pathogenic *Cryptococcus* species complex. (**a** and **b**) Ire1 plays a role in regulating virulence in an Hxl1-independent manner. *G. mellonella* in the final instar stage were infected with each *C. neoformans* strain and further incubated at 37 °C (**a**) or 25 °C (**b**). Percentage of survival (%) was monitored for 2 weeks post-infection. The survival curve was statistically analysed by log-rank (Mantel-Cox) test. At 25 °C, *P* = 0.003 for WT vs. *ire1*Δ mutant (YSB552), and *P* > 0.05 for WT vs. *hxl1*Δ mutant (YSB723). At 37 °C, *P* < 0.0001 for H99 vs. *ire1*Δ mutant and for H99 vs. *hxl1*Δ mutant. Strain information: WT (H99), *ire1*Δ mutant (YSB552), *ire1*Δ + *IRE1* (YSB1000), *hxl1*Δ mutant (YSB723), and *hxl1*Δ + *HXL1* (YSB762) (**c**) A/Jcr mice were infected with 5 × 10^4^ cells of WT (R265), *ire1*Δ mutant (YSB1889), *ire1*Δ + *IRE1* strain (YSB3158)*, hxl1*Δ mutant (YSB1891), and *hxl1*Δ + *HXL1* strain (YSB2957) by intranasal instillation. Survival was monitored for 60 days post-infection. The survival curve was statistically analysed by log-rank (Mantel-Cox) test. *P* = 0.0001 for WT vs. *ire1*Δ mutant and WT vs. *hxl1*Δ mutant; *P* = 0.8404 for WT vs. *ire1*Δ + *IRE1*; *P* = 0.5435 for WT vs. *hxl1*Δ + *HXL1*; *P* = 0.0002 for *ire1*Δ vs. *ire1*Δ + *IRE1*; and *P* = 0.0013 for *hxl1*Δ vs. *hxl1*Δ + *HXL1*. (**d**) The colony forming unit (CFU) count of *Cryptococcus* cells per gram of lung tissue was determined from sacrificed mice infected with WT (R265), *ire1*Δ mutant (YSB1889), *ire1*Δ + *IRE1* strain (YSB3158)*, hxl1*Δ mutant (YSB1891), and *hxl1*Δ + *HXL1* strain (YSB2957) by intranasal instillation 14 days post-infection.

Our previous study reported that both Ire1 and Hxl1 of the UPR pathway are indispensable for the virulence of *C. neoformans*^24^. We initially hypothesized that avirulence of both *ire1*Δ and *hxl1*Δ mutants in *C. neoformans* results from their severe growth defects at host physiological temperature (37 °C). However, it is still possible that a non-thermotolerance-related phenotype of the UPR mutants may contribute to their virulence defects. To address this question, the wax moth *Galleria mellonella* model for *Cryptococcus* infection provides a unique opportunity because the host can be incubated at 25 °C or 37 °C for virulence testing^34^. Similar to our previous data in the murine model of systemic cryptococcosis^24^, both *C. neoformans ire1*Δ and *hxl1*Δ mutants were avirulent in *G. mellonella* when the infected insect was incubated at 37 °C (Fig. 6a). Notably, however, the *C. neoformans ire1*Δ mutant, but not the *hxl1*Δ mutant, exhibited a complete lack of virulence in the insect incubated at 25 °C (Fig. 6b). This finding strongly suggests that thermotolerance-independent phenotypic traits significantly contribute to the avirulence of the *C. neoformans ire1*Δ mutant. In contrast to the *C. neoformans* strains, the *G. mellonella* model could be not used for testing the virulence of *C. deneoformans* and *C. deuterogattii* UPR mutants because wild-type JEC21 and R265 strains could not kill the insect even at 37 °C (data not shown).

To address the role of the UPR pathway in the virulence of *C. deuterogattii*, we employed a murine model of systemic cryptococcosis, as previously published^21^. Based on our observations from the insect killing assay with *C. neoformans* UPR mutants, we hypothesized that the *C. deuterogattii ire1*Δ mutant, but not the *hxl1*Δ mutant, might exhibit avirulence. As expected, the mice infected with the *C. deuterogattii ire1*Δ mutant did not show any signs of illness (monitored up to 60 days), whereas those infected with the wild-type R265 strain and the complemented strains became moribund within 20 days of infection (Fig. 6c). Surprisingly, however, the *C. deuterogattii hxl1*Δ mutants also did not cause illness in any mice (Fig. 6c), indicating that Hxl1 is still required for the virulence of *C. deuterogattii*.

To examine whether the avirulence of *C. deuterogattii ire1*Δ and *hxl1*Δ mutants is caused by reduced cell survival similar to *C. neoformans ire1*Δ and *hxl1*Δ mutants^24^, we measured fungal burden in the lungs and brains recovered from mice at day 14 post infection. The lung tissue recovered from mice infected with the *C. deuterogattii ire1*Δ mutant showed a very reduced burden (less than 400 CFU/g of tissue) compared to that from mice infected with wild-type and the complemented strain (more than 10^7^ CFU/g of tissue) (Fig. 6d). This is not a surprising result because the *C. deuterogattii ire1*Δ mutant cannot grow at 37 °C. Notably, mice infected with the *C. deuterogattii hxl1*Δ mutant, which did not show significant growth defects at 37 °C, also exhibited decreased fungal burden (less than 12,000 CFU/g of tissue) in the lungs (Fig. 6d). Even at day 64 post infection, the lungs of mice infected with the *hxl1*Δ mutant still showed low fungal burden (less than 33,000 CFU/g of tissue; Supplementary Table 1). As previously reported, a significant fungal burden was not observed in the brain tissue recovered from all infected mice (less than 3,000 CFU/g of tissue), owing to the lungs being the major target tissue of *C. deuterogattii* infection^17^.

**Figure 7:**
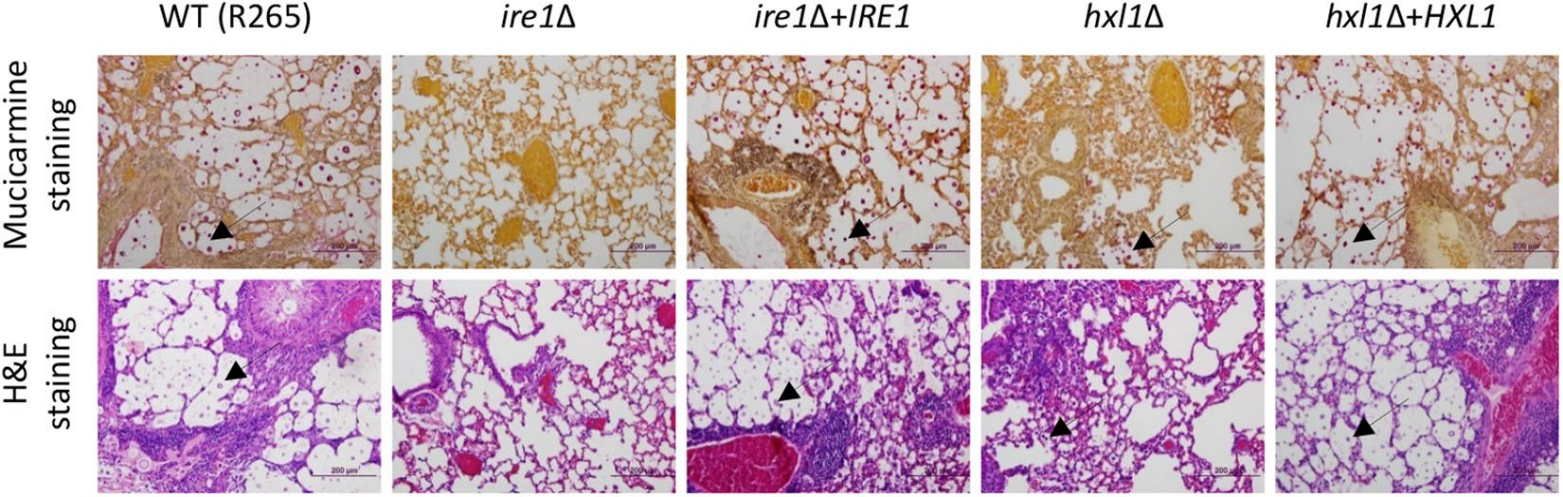
Histopathological analysis of *C. deuterogattii* UPR mutants in a murine model of systemic cryptococcosis. Mucicarmine-stained sections from wild-type R265 strain-infected mice showed extensive accumulation of fungi expanding and distending alveolar spaces. H&E-stained sections from R265-infected mice showed marked inflammatory changes. Photomicrographs of lung sections from *ire1*Δ mutant strain-infected mice displayed a lack of mutant cells and no inflammation in either airways or the parenchyma. Photomicrographs of sections from *ire1*Δ + *IRE1* or *hxl1*Δ + *HXL1* complemented strain-infected mice showed identical patterns of fungi and inflammation to those of R265-infected mouse. Photomicrographs of lung sections from mice infected with the *hxl1*Δ mutant, stained with mucicarmine, exhibited focal aggregations of fungi. H&E-stained sections from mice infected with the *hxl1*Δ mutant showed mild inflammatory changes. The arrow indicates *C. deuterogattii* strains in the lung tissue stained with mucicarmine or H&E. The scale bar indicates 200 μm.

Histopathological analysis of the infected lung and brain tissues further supported the observations made in the survival and fungal burden assays (Fig. 7). Each of the mice within the groups had similar histopathological findings. Mice infected with the wild-type R265 strain had marked expansion of alveolar spaces with numerous fungi (Fig. 7, upper panel in R265 lane). These fungus-laden regions of distended pulmonary parenchyma were associated with severe inflammatory changes consisting of angiocentric and bronchocentric accumulation of a pleocellular inflammatory infiltrate (Fig. 7, lower panel in R265 lane). Airway inflammation was severe within areas of high fungal burden, and bronchiolar epithelial hypertrophy/hyperplasia was marked. Neutrophils were present in the epithelium of large airways, and fungi and copious mucus were present within the lumens. No significant lesions or fungi were noted in the brain sections, supporting the finding that no significant fungal burden of the R265 strain was observed in the brain (data not shown). The *ire1*Δ + *IRE1* and *hxl1*Δ + *HXL1* complemented strains exhibited similar findings to the wild-type strain. In stark contrast to wild-type and complemented strains, no *ire1*Δ mutant cells were noted in mucicarmine-stained brain or lung sections (Fig. 7, upper panel in *ire1*Δ mutant lane; data not shown), and the lungs were free of inflammatory changes (Fig. 7, lower panel in *ire1*Δ mutant lane). In the case of mice infected with the *hxl1*Δ mutant, there were small focal areas where there were multiple contiguous alveolar spaces that contained fungi (Fig. 7, upper panel in *hxl1*Δ mutant lane). Pulmonary inflammation in these areas was mild and consisted of pleocellular inflammatory infiltrates, mild perivascular lymphoid cuffing, and mild bronchiolar epithelial hypertrophy (Fig. 7, lower panel in *hxl1*Δ mutant lane). Taken together, the results indicate that both Ire1 and Hxl1 of the UPR pathway are essential for virulence in *C. deuterogattii*.

### *C. deuterogattii* Hxl1 is involved in organic peroxide stress resistance in an Ire1-independent manner

**Figure 8:**
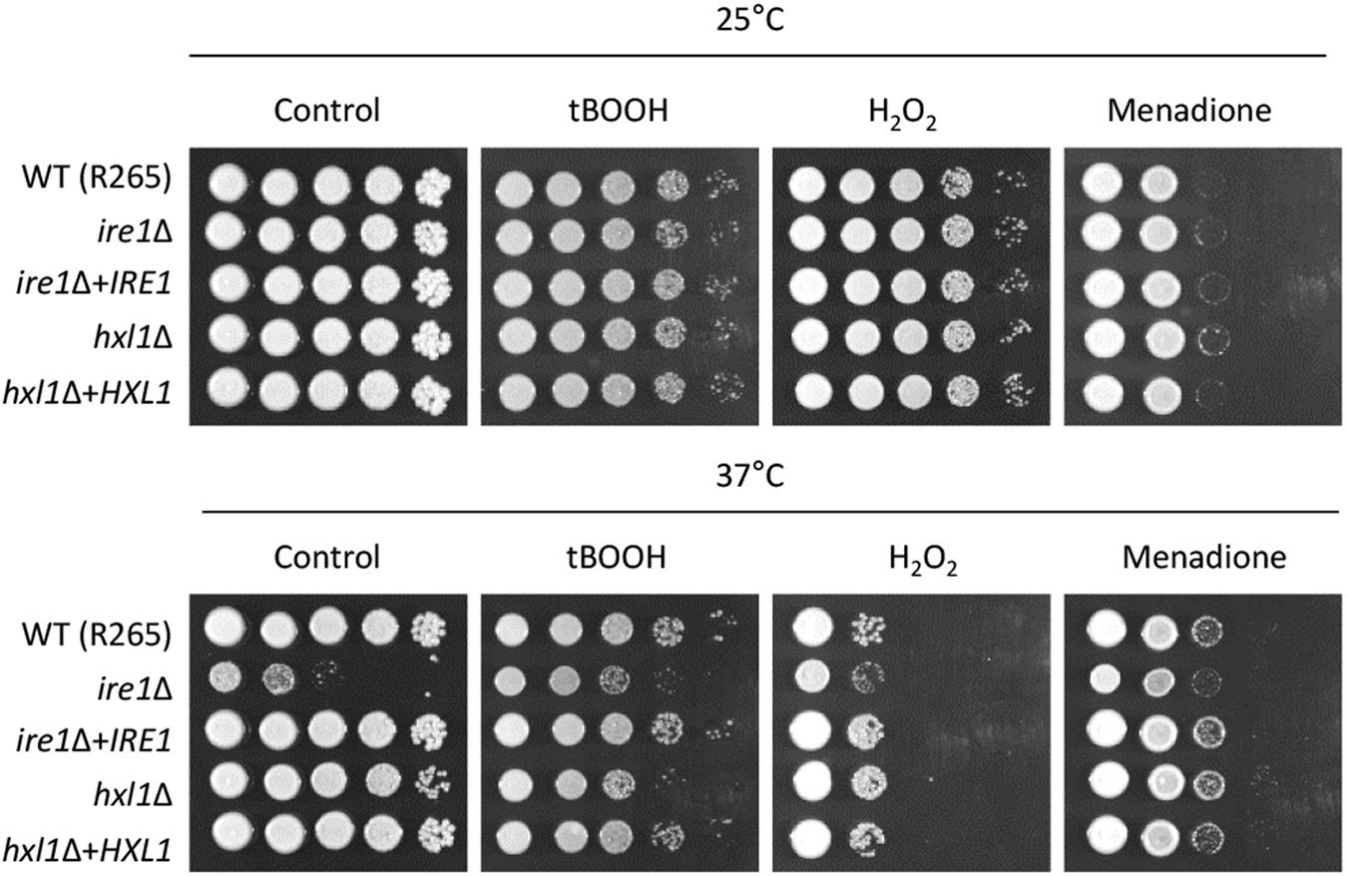
*C. deuterogattii* controls organic peroxide stress in an Hxl1-dependent manner at host physiological temperature. *C. deuterogattii* strains were incubated in liquid YPD medium at 30 °C overnight. Cells were serially diluted and spotted onto YPD medium containing the indicated concentrations of oxidative stress inducers [tBOOH (*tert*-butyl hydroperoxide, 0.5 mM), H_2_O_2_ (hydrogen peroxide, 3.5 mM), and menadione (0.03 mM)]. Plates were further incubated at 25 °C or 37 °C for 1–3 days and photographed daily.

The animal study revealed that Hxl1 contributes to the virulence of *C. deuterogattii* in a thermotolerance-independent manner. As *Cryptococcus* is exposed to reactive oxygen species (ROS) generated by the host immune system, we examined the possibility that Hxl1 may contribute to oxidative stress resistance in *C. deuterogattii*. To address this question, we examined the susceptibility of *C. deuterogattii* UPR mutants to the following oxidative stress agents: hydrogen peroxide (H_2_O_2_), *tert*-butyl hydroperoxide (tBOOH; organic peroxide), and menadione (a superoxide generator). Notably, both *C. deuterogattii ire1*∆ and *hxl1*∆ mutants were as resistant to the oxidative stresses as the wild-type strain at 25 °C (Fig. 8). At 37 °C, however, the *hxl1*∆ mutant exhibited very weakly increased susceptibility to tBOOH, but not to H_2_O_2_ or menadione (Fig. 8). As the *C. deuterogattii ire1*∆ mutant did not show any increased susceptibility to tBOOH at 37 °C compared to control (Fig. 8), the role of Hxl1 in the organic peroxide resistance appeared to be Ire1-independent in *C. deuterogattii*. This phenomenon suggests that the role of Hxl1 in resistance to certain oxidative stress, in addition to its role in ER stress response and adaptation, might be one of contributing factors for the avirulence of the *C. deuterogattii hxl1*∆ mutant.

## Discussion

**Figure 9:**
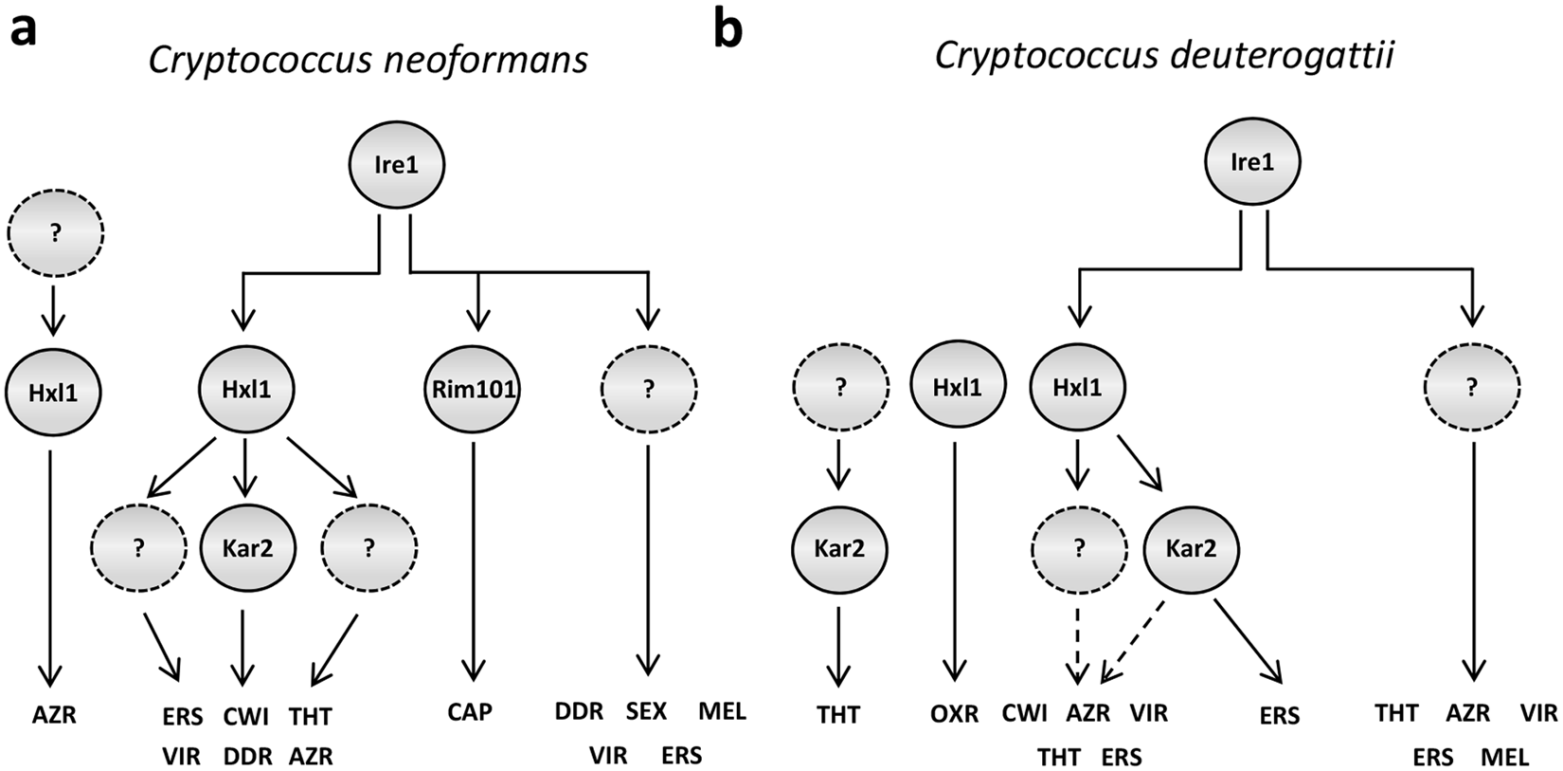
The proposed model for UPR pathways of the pathogenic *Cryptococcus* species complex. (**a**) *C. neoformans* Ire1 controls environmental stress responses, differentiation, and virulence in Hxl1-dependent and -independent manners. The Ire1-Hxl1-dependent UPR pathway governs responses to ER and cell wall/membrane stress, azole drug resistance, and thermotolerance through controlling expression levels of *KAR2*. In capsule production, the transcription factor Rim101 acts as a downstream factor of Ire1. The unidentified downstream factors of Ire1 regulate DNA damage response, sexual differentiation, and melanin production. (**b**) *C. deuterogattii* (R265) Ire1 regulates environmental stress responses and virulence in Hxl1-dependent and -independent manners. The Ire1-Hxl1-dependent UPR pathway mainly controls cell wall/membrane integrity, azole drug resistance, and ER stress. Ire1 mostly regulates thermotolerance, and melanin synthesis. ERS, ER stress; CWI, cell wall/membrane integrity; THT, thermotolerance; VIR, virulence; DDR, DNA damage response; AZR, azole drug resistance; CAP, capsule production; SEX, sexual differentiation; MEL, melanin synthesis; OXR, oxidative stress response.

The UPR pathway has been characterized in diverse fungi including *Saccharomyces cerevisiae*, *Cryptococcus neoformans*, *Candida albicans*, *Candida glabrata*, *Aspergillus fumigatus*, *Alternaria brassicicola*, and *Ustilago maydis*^24,26,35–39^. In this study, we functionally characterized *in vitro* and *in vivo* roles of the UPR pathway in *C. deuterogattii* and compared them with those in *C. neoformans* (Fig. 9). Here, we demonstrated that the UPR pathway has evolutionarily conserved roles in the pathogenic *Cryptococcus* species complex. The most evident conserved role is ER stress response and adaptation. In response to ER stress, the unconventional, spliceosome-independent splicing event of *HXL1* mRNA and induction of *KAR2* occurred in Ire1-dependent manners in all studied species. Other conserved roles include azole drug resistance, cell wall integrity, thermotolerance, and virulence. Therefore, it seems clear that the UPR pathway has evolved as a core signalling pathway for stress response and virulence of the pathogenic *Cryptococcus* species complex.

Regardless of its conserved roles, several notable functional divergences of Ire1 and Hxl1 were observed. The most notable difference was the different roles of Ire1 and Hxl1 in the thermotolerance of *C. deuterogattii*. Deletion of *HXL1* did not significantly affect the growth of *C. deuterogattii* at 37 °C, whereas the *ire1*Δ mutant could not grow at the host physiological temperature. This is in contrast to the finding that deletion of *HXL1* causes even more severe growth defects at 37 °C than *IRE1* in *C. neoformans*. These findings suggest that the *C. deuterogattii* UPR pathway might control thermotolerance in a fashion different than it does in *C. neoformans*. Supporting this, we found that the expression level of *PMT1* was similar among R265 and UPR mutants upon temperature upshift, although it is significantly downregulated in the *C. neoformans hxl1*Δ mutant^24^. Ire1 appears to mainly regulate the thermotolerance of *C. deuterogattii* through activation of other unknown transcription factors. In fact, we previously observed that complementation of the *ire1*Δ mutant with a spliced version of *HXL1* (induced active form) only partially restores the growth of *C. neoformans* at 37 °C^24^, suggesting that other transcription factors along with Hxl1 mediate its Ire1-dependent thermotolerance. It is probable that such transcription factors could play a major role in the UPR pathway-mediated thermotolerance in *C. deuterogattii*. Supporting this, the expression of *KAR2* is positively regulated by Ire1 and Hxl1 in *C. neoformans* upon temperature upshift^24^. However, we found that *C. deuterogattii* Ire1 and Hxl1 do not regulate *KAR2* induction upon temperature upshift. In addition to thermotolerance, azole drug resistance patterns of UPR mutants are different within the pathogenic *Cryptococcus* species complex. In both *C. neoformans* and *C. deneoformans*, *hxl1*∆ mutants are more susceptible to azole drugs than *ire1*∆ mutants, which is opposite to the phenotype of *C. deuterogattii* UPR mutants. In addition to Hxl1, other transcription factors could also modulate the UPR-mediated azole drug resistance in *C. deuterogattii*. Such Ire1-dependent transcription factors involved in thermotolerance and azole drug resistance remain to be further identified and characterized in future studies.

This type of functional divergence in conserved signalling components appears to be extensive in the pathogenic *Cryptococcus* species complex. One example of this is found in the catalytic subunits Pka1 and Pka2 of PKA in the cAMP pathway. In *C. neoformans*, Pka1 regulates melanin and capsule production, whereas Pka2 is not involved in virulence factor formation^18^. In *C. deneoformans*, however, Pka2, but not Pka1, plays a role in virulence factor production^19^. In *C. deuterogattii*, Pka1 and Pka2 share roles in capsule production and mating responses, and Pka2, but not Pka1, retains its function in melanin biosynthesis^20^. As another example, *C. neoformans* and *C. deuterogattii* calcineurin orthologs have divergent roles in controlling Ca^2+^ and Li^2+^ homeostasis and fluconazole tolerance^21^. This functional divergence of evolutionarily conserved signalling components in several core virulence-regulating signalling pathways may contribute to the competitive fitness of the pathogenic *Cryptococcus* species complex in diverse environmental and host niches and to different infectivity and virulence potentials.

In this study, we found that the role of the UPR pathway in azole drug resistance is conserved in the pathogenic *Cryptococcus* species complex, but in a way different from other human fungal pathogens such as *A. fumigatus* and *C. glabrata*^36–38^. Askew *et al*. reported that UPR components such as HacA and IreA play critical roles in antifungal drug resistance of *A. fumigatus*^37,38^. Transcriptome analysis revealed that expression levels of many ergosterol biosynthesis genes including *ERG11* are reduced in both *hacA* and *ireA* mutants. Supporting this, the ergosterol content itself is also decreased in the *A. fumigatus* UPR mutant^38^. In contrast, basal and fluconazole-mediated induced expression levels of *ERG11* in all UPR mutants of the pathogenic *Cryptococcus* species complex were not different from wild-type levels, indicating that the UPR pathway does not directly regulate ergosterol biosynthesis. Instead, the UPR-mediated cell membrane stability appears to contribute to azole drug resistance of the *Cryptococcus* species at least partly, because azole treatment eventually perturbs cell membrane integrity. Similar to the case in *Cryptococcus*, perturbation of *IRE1* alone does not affect azole drug susceptibility in *C. glabrata*^36^, indicating that the UPR pathway is not a direct regulator of ergosterol biosynthesis in these pathogenic yeasts.

As the most important finding in this study, we showed that ER stress response and adaptation itself are critical for the virulence of the pathogenic *Cryptococcus* species. This has been a challenging question because impairment of the UPR pathway leads to pleiotropic effects, including the inability to grow at 37 °C, which is the most obvious virulence determinant for *C. neoformans*. In this study, we provided two pieces of evidence showing that the UPR pathway contributes to the virulence of the pathogenic *Cryptococcus* species in a thermotolerance-independent manner. First, the *C. neoformans ire1*Δ mutant did not kill the wax moth *G. mellonella* when the insect was incubated at 25 °C. The finding that the *C. neoformans hxl1*Δ mutant exhibited wild-type levels of virulence in the *G. mellonella* model at 25 °C suggests that proliferation regulated by Hxl1 at the host physiological temperature is an essential factor contributing to virulence of *C. neoformans*. Second, the *C. deuterogattii hxl1*Δ mutant, which did not show significant growth defects at 37 °C, is avirulent in the murine model of systemic cryptococcosis. Although the *C. deuterogattii hxl1*Δ mutant showed a minor growth defect in the presence of tBOOH at 37 °C, the most notable phenotype of the *hxl1*Δ mutant was high susceptibility to ER stress agents such as TM (an *N*-glycosylation inhibitor) and DTT. Therefore it is likely that ER stress response and adaption itself are critical for the virulence of *C. deuterogattii*. Previous studies revealed that *N*-glycosylation, a posttranslational modification, occurs in the ER and affects fungal pathogenesis^40,41^. Furthermore, *O*-mannosylation, which is initiated in the ER, also contributes to fungal virulence^42,43^. Specifically, the deletion of *O*-mannosyltransferases *PMT1* and *PMT4* attenuates virulence in *C. neoformans*^44,45^. Our finding that expression of lipid-linked *N*-oligosaccharyltransferase genes including *ALG7*, *WBP1*, and *OST1* is regulated by the UPR pathway supports that Hxl1-mediated ER stress response and adaptation is important for the virulence of *C. deuterogattii*.

In conclusion, our data suggest that the UPR pathway in *C. deuterogattii* plays evolutionarily conserved and divergent roles in stress responses and virulence compared to *C. neoformans*.

## Methods

### Ethics statement

Animal studies were conducted in the Division of Lab Animal Research at Duke University according to guidelines set forth by the United States Animal Welfare Act and the Duke Institutional Animal Care and Use Committee. The methods were approved by the Duke IACUC under protocol A171-16-08.

### Strains and growth conditions

*C. neoformans, C. deneoformans*, and *C. deuterogattii* strains used in the study are listed in Supplementary Table 2 and were cultured on yeast extract-peptone-dextrose (YPD) medium. For capsule production assay, the agar-based Dulbecco’s modified Eagle’s medium (DMEM; Invitrogen, Carlsbad, CA) and Littman’s medium were used^19,33,46^. For melanin biosynthesis, Niger seed medium containing the indicated concentration of glucose was prepared as previously described^46^.

### Construction of *C. deuterogattii* R265 strain *ire1*Δ and *hxl1*Δ mutants

For disruption of *IRE1* or *HXL1* genes in the *C. deuterogattii* R265 strain, information regarding the *IRE1* or *HXL1* genomic structure and sequences was obtained from the *C. deuterogattii* genomic database (http://www.broadinstitute.org/annotation/genome/cryptococcus_neoformans_b/MultiHome.html). Primers used for the amplification of 5′- and 3′-flanking regions of *IRE1* or *HXL1* genes in the *C. deuterogattii* R265 strain are listed in Supplementary Table 3. M13Fe and M13Re primers were used for amplifying the Nar^r^ dominant selectable marker. The *IRE1* and *HXL1* gene disruption cassettes were generated by double-joint PCR (DJ-PCR), as previously described^47^. Each gel-extracted gene disruption cassette was biolistically inserted into the *C. deuterogattii* R265 strain. Stable transformants were selected on YPD medium containing nourseothricin and screened by diagnostic PCR. To verify correct genotypes of *ire1*Δ and *hxl1*Δ mutants in R265 strains, Southern blot analysis was performed (Supplementary Fig. 7)^48^.

### Construction of *IRE1* and *HXL1* complemented strains of the *C. deuterogattii* R265 strain

To verify the phenotypes observed in R265 *ire1*Δ and *hxl1*Δ mutants, we constructed corresponding complemented strains as follows. For construction of *IRE1* and *HXL1* complemented strains of the R265 strain, PCR fragments containing 5′- and 3′-flanking regions and open reading frames (ORFs) of *IRE1* or *HXL1* genes were amplified with primers B5808/B5809 and B5803/B5804, respectively. The amplified PCR products were cloned into the plasmid pTOP-V2 to produce the plasmids pTOP-IRE1 (YSBE542) and pTOP-HXL1 (YSBE543). After confirming the DNA sequence with no errors, the cloned genes were subcloned into the plasmid pJAF12 to produce the plasmids pJAF12-IRE1 (YSBE544) and pJAF12-HXL1 (YSBE545). The designated restriction enzyme-digested plasmids (NdeI for pJAF12-IRE1 (YSBE544) and PmlI for pJAF12-HXL1 (YSBE545)) were linearized and biolistically inserted into the *ire1*Δ (YSB1889) and *hxl1*Δ (YSB1891) mutants, respectively. The re-integration of *IRE1* and *HXL1* into their native loci was confirmed by diagnostic PCR with the primer pairs B4644/B4646 and B4638/B4640, respectively.

### Stress sensitivity test

Each pathogenic *Cryptococcus* strain was incubated overnight at 30 °C in liquid YPD medium, washed, serially diluted (dilutions from 1 to 10^−4^) with dH_2_O, and spotted (3 μL) onto solid YPD medium containing the indicated concentrations of stress inducers. To test ER and cell wall/membrane stress, cells were spotted onto a solid YPD medium containing the indicated concentration of the ER stress inducers tunicamycin (TM) or dithiothreitol (DTT), cell wall stress inducers calcofluor white (CFW) or Congo red (CR), or cell membrane destabilizers sodium dodecyl sulphate (SDS) or fludioxonil (FDX). To determine thermotolerance, plates were incubated at 30 °C, 37 °C, and 39 °C. For the antifungal drug resistance test, cells were spotted onto a solid YPD medium containing the indicated concentrations of the azole drugs fluconazole (FCZ), ketoconazole (KCZ), and itraconazole (ICZ). Cells were further incubated at 30 °C for 4 or 5 days and photographed daily. For oxidative stress, *C. deuterogattii* cells were cultured at 30 °C in liquid YPD medium, washed, serially diluted (dilutions from 1 to 10^−4^) with dH_2_O, and spotted (3 μL) onto solid YPD medium containing the indicated concentration of oxidative stress inducers. Cells were further incubated at 25 °C or 37 °C for 1–3 days and photographed daily.

### Total RNA isolation, RT-analysis, and quantitative RT-PCR

Total RNA was isolated with the TRIzol reagent as previously described^49^. For cDNA synthesis, the total RNA amount was adjusted to 5 μg with DEPC-treated water, to which 1 μL of 5 μg/μL oligo (dT)-pdN6 was added, and the mixture was incubated at 70 °C for 10 min and then placed on ice for 10 min. Next, 15 μL of the cDNA synthesis mixture including reverse transcriptase (Thermo Fisher Scientific, Waltham, MA), RNase inhibitor (Intron), and 10 mM dNTP was added and further incubated at 42 °C for 2 h. After incubation, the mixture was heat-inactivated at 65 °C for 15 min. To monitor *HXL1* splicing events, RT-PCR of *HXL1* and *ACT1* was performed with gene-specific primers listed in Supplementary Table 3. For quantitative RT-PCR analysis, expression levels of *ERG11*, *ERG3*, *KAR2, DER1, ALG7, PMT1, PMT4, WBP1, OST1, SEC61, ERV29, SOD2, PPS1*, and *CHS2* were measured with gene-specific primers listed in Supplementary Table 3 on a MyiQ2 Real-Time PCR detection system. For qRT-PCR, target gene expression levels were normalized using *ACT1* expression levels as controls.

### Melanin and capsule test

For the capsule and melanin assay, cells were incubated overnight (16 h) at 30 °C in a liquid YPD medium. For melanin assay, 3 μL of cells were spotted on agar-based Niger seed medium, which contained the indicated concentration of glucose (0.1%). The plates were incubated at 30 °C, monitored daily, and photographed using SPOT insight digital camera (Diagnostic Instruments Inc., Sterling Heights, MI). For capsule production assay, cells were spotted onto agar-based DMEM or cultured in liquid Littman’s medium at 30 °C for 2 days. For visualization of capsule synthesis, cells scraped from agar-based DMEM or cultured in liquid Littman’s medium were resuspended in PBS, stained with India ink (BACTIDROP^TM^; Remel, San Diego, CA), and observed under the Olympus BX51 microscope equipped with a SPOT Insight digital camera (Diagnostic Instruments Inc.). For quantitative measurement of capsule, cells were collected from liquid Littman medium. The relative capsule diameter was determined by the following equation (1): (*D*_*w*_–*D*_*c*_) × *D*_*w*_, where *D*_*w*_ and *D*_*c*_ indicate the diameter of the whole cell body and the diameter of the cell body, respectively^46^.

### *Galleria mellonella* infection assay and virulence assay

The *G. mellonella* infection assay was performed by following the previously described method with minor modifications^34^. Fifteen *G. mellonella* caterpillars in the final instar stage (Vanderhorst, Inc., St Marys Ohio USA) were used per each group. *C. neoformans* strains were grown in liquid YPD media for 16 h at 30 °C and then washed three times with PBS. Cells were resuspended in PBS and counted using a haemocytometer. The caterpillars were inoculated with 4 μL of 1 × 10^6^ cells/mL using Hamilton syringes equipped with 10-μL needles. PBS was injected as a negative control. After injection, caterpillars were incubated at 25 °C or 37 °C and monitored daily. The survival curve was illustrated by Prism 6 (GraphPad Software, La Jolla, CA) and statistically analysed by log-rank (Mantel-Cox) test.

### Mouse study

Five- to six-week-old female A/Jcr mice (Charles River, Wilmington, MA) were used for this study. Strains were grown in YPD broth for 24 h at 24 °C and washed twice in sterile PBS (Sigma-Aldrich, St. Louis, MO). Cells were counted using a haemocytometer, and the final inoculum was adjusted to 50000 CFU/50 μL of PBS. This volume was administered to each mouse. Groups of 10 mice for each strain were anesthetized with isoflurane and infected via intranasal instillation. Mice were monitored each day as described in the approved protocol and euthanized by CO_2_ inhalation upon reaching humane endpoints. The survival curve was created using GraphPad Prism software, and *P* values of < 0.05 were considered significant. Fungal burden analysis of brains and lungs was performed at 14 days post-infection. Tissue samples were homogenized using a Biospec bead beater and steel beads (Qiagen, Hilden, Germany). Homogenates were serially diluted, and 100 μL of the homogenates were plated on YPD plates with chloramphenicol and ampicillin. Plates were incubated at 24 °C for 48–72 h, after which the burden (CFU/g of tissue) was measured. For histopathology, each organ was cut in half and fixed in 10% buffered formalin solution (Thermo Fisher Scientific), embedded in paraffin and sectioned at 5 μm. Histopathological changes were assessed on H&E-stained sections by a board-certified veterinary pathologist without knowledge of the group. Mucicarmine-stained sections were evaluated to visualize fungi.

## Electronic supplementary material

Supplementary information
