# Supplementary material for: Evolutionarily Conserved and Divergent Roles of Unfolded Protein Response (UPR) in the Pathogenic Cryptococcus Species Complex
Source: Sci Rep. 2018 May 25;8:8132. doi: 10.1038/s41598-018-26405-5 (PMC5970146; doi:10.1038/s41598-018-26405-5)
Supplement: Supplementary file 1 — Supplementary information [file 41598_2018_26405_MOESM1_ESM.pdf]

Supplementary Information

Evolutionarily Conserved and Divergent Roles of Unfolded Protein Response (UPR) in the Pathogenic *Cryptococcus* Species Complex

Kwang-Woo Jung,[1†] Kyung-Tae Lee,[1]Anna F. Averette,[2] Michael J. Hoy,[2] Jeffrey Everitt,[3] Joseph Heitman,[2] and Yong-Sun Bahn[1*]

[1] Department of Biotechnology, Yonsei University, Seoul 03722, Republic of Korea
[2] Department of Molecular Genetics and Microbiology, Medicine, and Pharmacology and Cancer Biology, Duke University Medical Center, Durham, North Carolina 27710, USA
[3] Department of Pathology, Duke University Medical Center, Durham, North Carolina 27710, USA

[†] Present address: Research Division for Biotechnology, Korea Atomic Energy Research Institute, Jeongeup 56212, Korea

Contents

• **Supplementary Figure 1**
• **Supplementary Figure 2**
• **Supplementary Figure 3**
• **Supplementary Figure 4**
• **Supplementary Figure 5**
• **Supplementary Figure 6**
• **Supplementary Figure 7**
• **Supplementary Table 1**
• **Supplementary Table 2**
• **Supplementary Table 3**

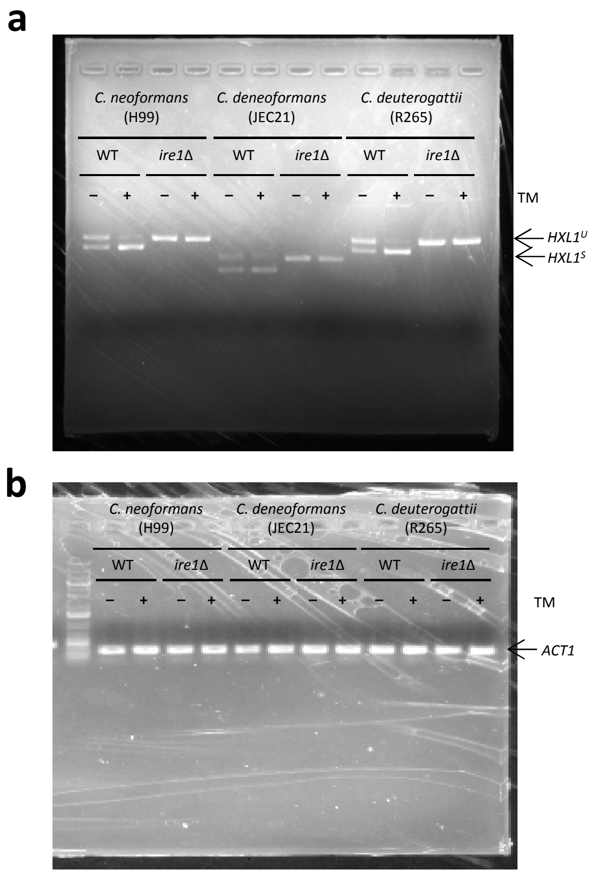

Supplementary Figure 1. RT-PCR analysis of *HXL1* splicing in response to ER stress. (a and b) The original images of RT-PCR analysis of *HXL1* splicing and *ACT1* in *C. neoformans*, *C. deneoformans*, and *C. deuterogattii* in response to ER stress.

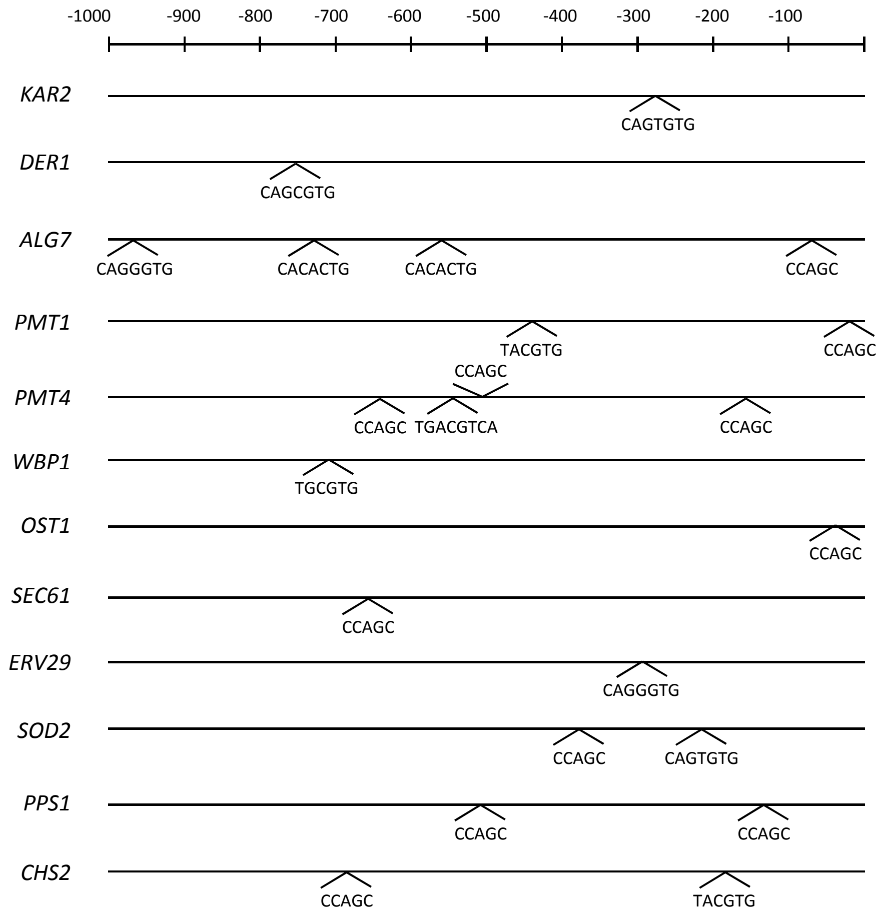

Supplementary Figure 2. Putative UPR genes of *C. deuterogattii* harbouring the UPRE (CAGNGTG) and UPRE-like (TGACGTCA, CCAGC, CGTGTCGG, or TACGTG) sequences. A set of promoter sequence (-1000 ~ -1) of putative UPR regulated genes of *C. deuterogattii* were from the FungiDB (www.fungidb.org).

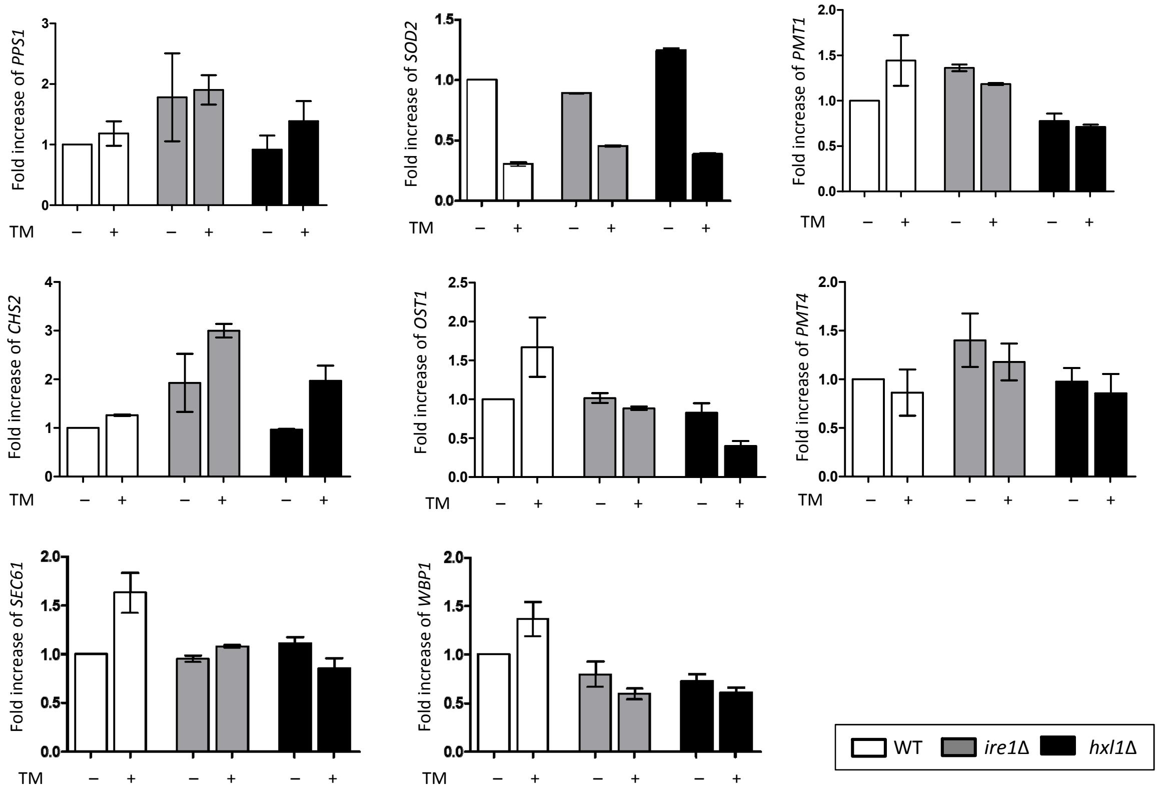

Supplementary Figure 3. qRT-PCR analysis of putative UPR-regulated genes upon ER stress. The expression levels of putative UPR-regulated genes were measured in WT, *ire1*Δ, and *hxl1*Δ mutants through qRT-PCR analysis. *C. deuterogattii* strains were grown in liquid YPD medium to mid-log phase (OD_{600}=0.6), exposed to TM (0.3 µg/mL) for 1 h, and harvested. The cDNA was synthesized from total RNA in cells treated or not treated with TM. Duplicate technical experiments with two biological samples were performed. Representative images from independent experiments for the target gene are shown. Error bars indicate standard deviation.

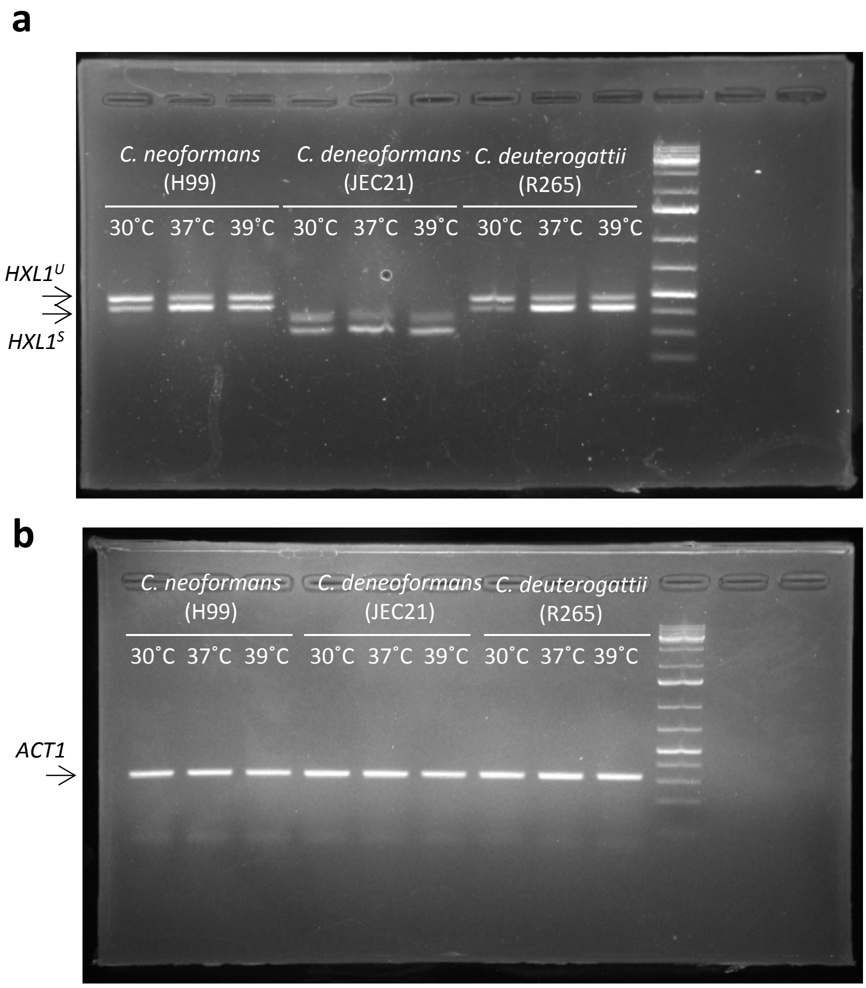

Supplementary Figure 4. RT-PCR analysis of *HXL1* splicing in high temperature conditions. (a and b) The original images of RT-PCR analysis of *HXL1* splicing and *ACT1* in *C. neoformans*, *C. deneoformans*, and *C. deuterogattii* in response to high temperature.

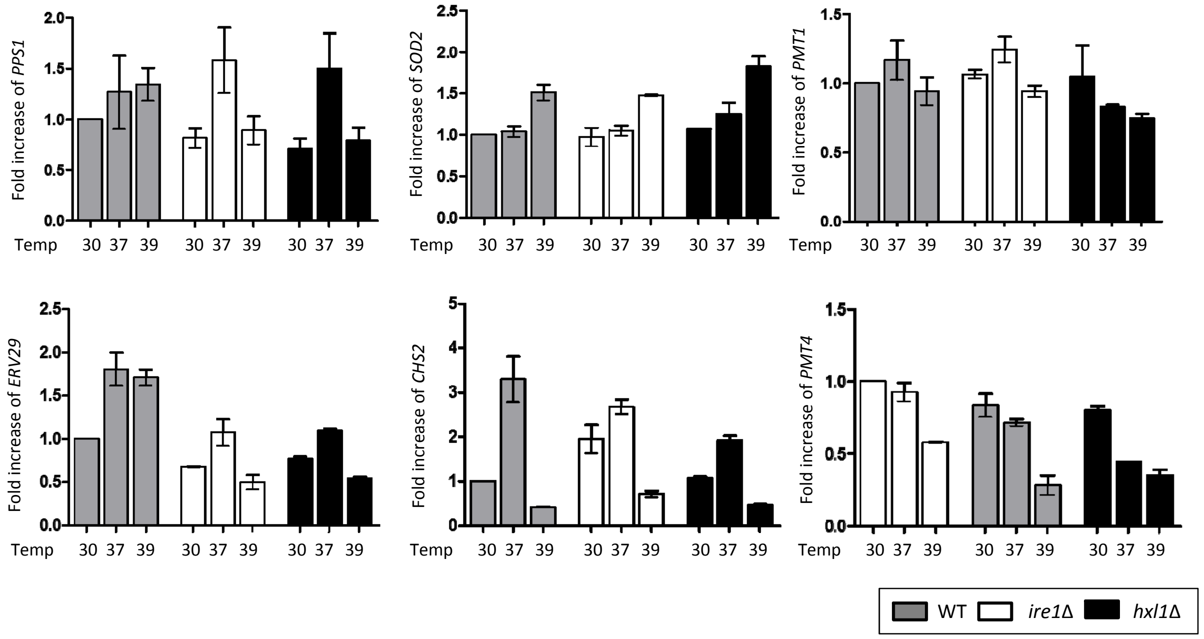

Supplementary Figure 5. qRT-PCR analysis of putative UPR-regulated genes upon temperature upshift. The expression levels of putative UPR-regulated genes were measured in WT, *ire1*Δ, and *hxl1*Δ mutants through qRT-PCR analysis. Duplicate technical experiments with three biological samples were performed. Representative images from independent experiments for the target gene are shown. Error bars indicate standard deviation.

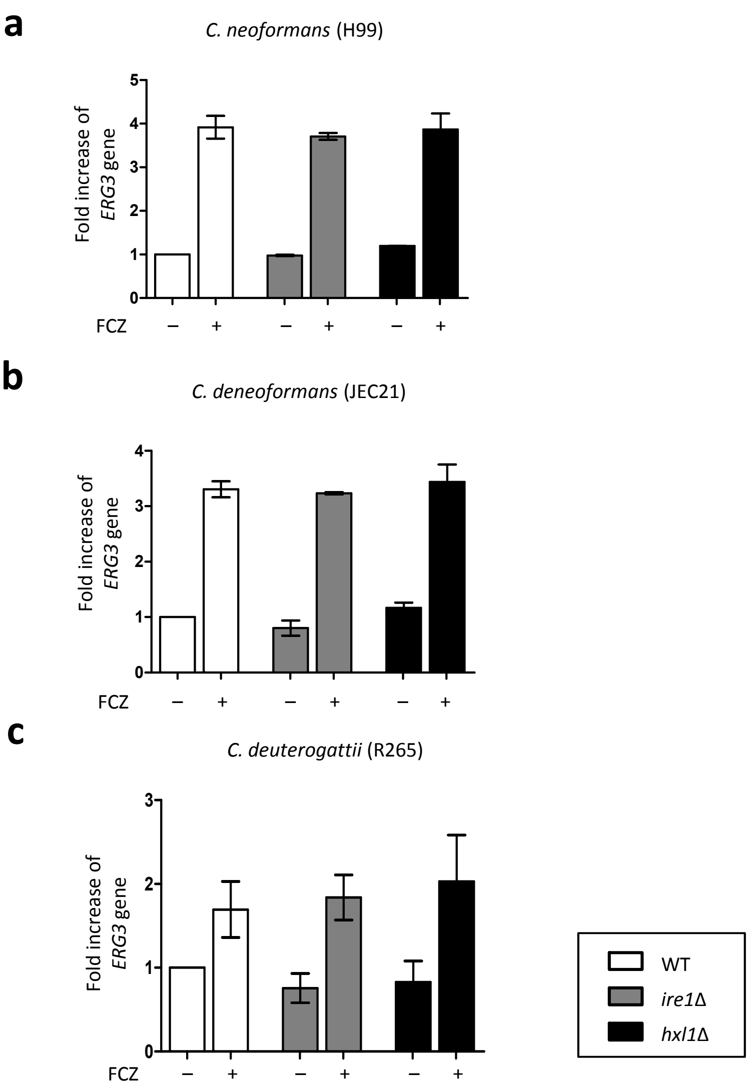

Supplementary Figure 6. The UPR pathways in the pathogenic *Cryptococcus* species complex are not involved in the induction of the *ERG3* gene in response to fluconazole treatment. The induction of *ERG3* was measured in WT, *ire1*Δ, and *hxl1*Δ mutants through qRT-PCR analysis. The cDNA was synthesized from total RNA in cells treated or not treated with fluconazole (10 µg/ml) for 90 mins. Duplicate technical experiments with two biological samples were performed. Representative images from independent experiments for the *ERG3* gene are shown. Error bars indicate standard deviation.

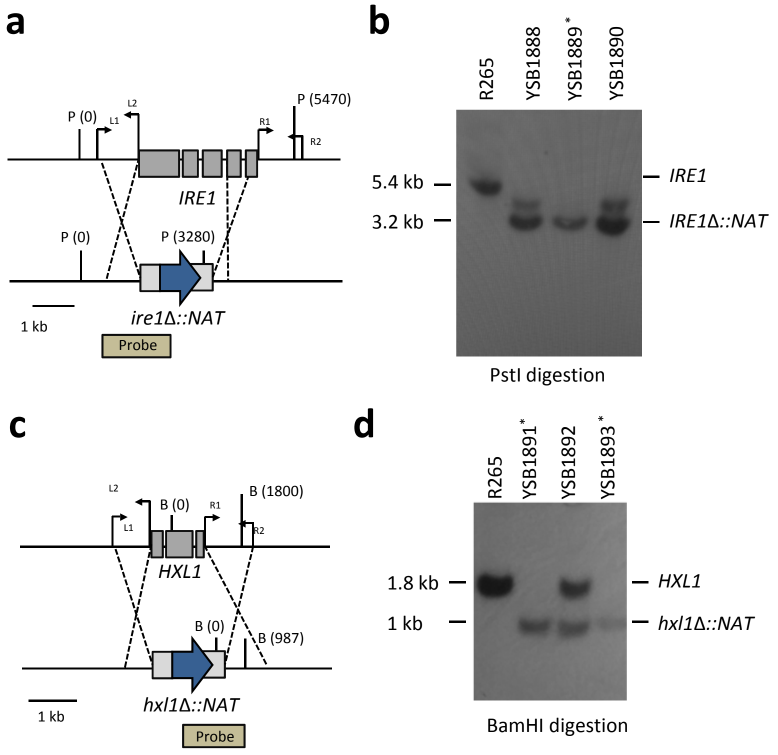

Supplementary Figure 7. Disruption of *C. neoformans IRE1* and *HXL1* in the *C. deuterogattii* R265. (a and c) A diagram of disruption of the *IRE1* gene and *HXL1* gene in *C. deuterogattii* R265. (b and d) The correct gene disruption was verified by Southern blot analysis using genomic DNAs digested with the indicated restriction enzyme. Strains marked with asterisks were used in this study.

Supplementary Table 1. Fungal burden data analysis in animals infected with *C. deuterogattii* strains.

Day[a]	Strain information	Mouse ID	CFU/g Lung	CFU/g Brain
14	R265	1	20900000	0
		2	19636363.64	0
		3	15038461.54	964.29
	*ire1*Δ	1	400	0
		2	166.67	0
		3	111.11	0
	*ire1*Δ + *IRE1*	1	14812500	941.18
		2	19205882.35	294.12
		3	27058823.53	0
	*hxl1*Δ	1	5055.56	921.05
		2	11333.33	3000
		3	45.45	0
	*hxl1*Δ + *HXL1*	1	46142857.14	0
		2	32026315.79	0
		3	26309523.81	0
50	*ire1*Δ	1	137.93	0
		2	590.91	428.57
		3	0	0
	*hxl1*Δ	1	56184.21	597.22
		2	137285.71	4542.86
		3	110666.67	2903.23
64	*ire1*Δ	1	51.28	14.29
		2	0	0
		3	32.26	13.16
		4	27.78	171.05
	*hxl1*Δ	1	13333.33	0
		2	33030.30	5500
		3	18000	175.68
		4	264.71	150

[a] Each organ was recovered from the mice infected with *C. deuterogattii* strains on the indicated day post infection.

Supplementary Table 2. Strains used in this study

Strain	Genotype	Parent	Reference
H99	*MATα*		[1]
YSB552	*MATα ire1Δ::NAT-STM#224*	H99	[2]
YSB1000	*MATα ire1Δ::NAT-STM#224 IRE1-NEO*	YSB552	[2]
YSB723	*MATα hxl1Δ::NAT-STM#295*	H99	[2]
YSB762	*MATα hxl1Δ::NAT-STM#295 HXL1-NEO*	YSB723	[2]
JEC21	*MATα*		[3]
YSB2886	*MATα ire1Δ::NAT-STM#273*	JEC21	[4]
YSB2030	*MATα hxl1Δ::NAT-STM#58*	JEC21	[4]
R265	*MATα*		[5]
YSB1889	*MATα ire1Δ::NAT-STM#273*	R265	This study
YSB3158	*MATα ire1Δ::NAT-STM#273 IRE1-NEO*	YSB1889	This study
YSB1891	*MATα hxl1Δ::NAT-STM#224*	R265	This study
YSB1893	*MATα hxl1Δ::NAT-STM#224*	R265	This study
YSB2957	*MATα hxl1Δ::NAT-STM#224 HXL1-NEO*	YSB1891	This study

Each *NAT-STM#* indicates the Nat^r marker with a unique signature tag.

Supplementary Table 3. Primers used in this study

Primer Name	Sequence	Comment
B79	TGTGGATGCTGGCGGAGGATA	Screening primer on *ACT1* promter
B1026	GTAAAACGACGGCCAGTGAGC	M13 forward (extended)
B1027	CAGGAAACAGCTATGACCATG	M13 reverse (extended)
B1454	AAGGTGTTCCCCGACGACGAATCG	NSL2
B1455	AACTCCGTCGCGAGCCCCATCAAC	NSR2
B4644	ACAGGATTAGTCTTGGGCG	*IRE1* – 5' screening primer (*C. deuterogattii*)
B4645	TCCTCAGAACACCTGTAACG	*IRE1* – left flanking primer 1 (*C. deuterogattii*)
B4646	TCACTGGCCGTCGTTTTACGAAGTATGTTTGCTGCCG	*IRE1* – left flanking primer 2 (*C. deuterogattii*)
B4647	CATGGTCATAGCTGTTTCCTGAAGGGTGTTTCCAAGAGGC	*IRE1* – right flanking primer 1 (*C. deuterogattii*)
B4648	AAACAGCCGCTCTCAACAC	*IRE1* – right flanking primer 2 (*C. deuterogattii*)
B4649	CGATTCCAAATGAGTCCAC	*IRE1* – probe primer 1 (for Southern blot, *C. deuterogattii*)
B5808	CGCGCGGCCGCGTTGACTGGATTTGCGTGGAA	*IRE1* left flanking primer 1 for cloning (*C. deuterogattii*)
B5809	CGCGCGGCCGCCGTGAGCAGCAAGTTGAAGGC	*IRE1* right flanking primer 1 for cloning *C. deuterogattii*)
B5810	ACATTGTGGAACCCTTGAG	*IRE1* sequencing primer 1 (*C. deuterogattii*)
B5811	TCTTGGTGTGAAGCCTGGTG	*IRE1* sequencing primer 2 (*C. deuterogattii*)
B5812	GAAGAGGAAGAACAAGTGCTG	*IRE1* sequencing primer 3 (*C. deuterogattii*)
B5813	GAGCGTTGATGGAGGTTAC	*IRE1* sequencing primer 4 (*C. deuterogattii*)
B5814	CTTTTTGTGTGACGCTTCG	*IRE1* sequencing primer 5 (*C. deuterogattii*)
B4638	CACTGACCCGATAGAACTTG	*HXL1* – 5' screening primer (*C. deuterogattii*)
B4639	GGGCTATTGTCACTTCTGG	*HXL1* – left flanking primer 1 (*C. deuterogattii*)
B4640	TCACTGGCCGTCGTTTTACGAGAAAGAAAGGAGACGGG	*HXL1* – left flanking primer 2 (*C. deuterogattii*)
B4641	CATGGTCATAGCTGTTTCCTGGGAGGAGAAGACAGATGACAG	*HXL1* – right flanking primer 1 (*C. deuterogattii*)
B4642	GGTCGTAATAGGATGGAGGC	*HXL1* – right flanking primer 2 (*C. deuterogattii*)
B5681	CTTTCACCTGGTCTTCCTG	*HXL1* – probe primer 1 (for Southern blot, *C. deuterogattii*)
B5803	CGCGCGGCCGCTAGCCCTCAATACCGACAC	*HXL1* left flanking primer 1 for cloning (*C. deuterogattii*)
B5804	CGCGCGGCCGCAAGGTCTCGGGGGATTATC	*HXL1* right flanking primer 1 for cloning *C. deuterogattii*)
B5805	GAATCATCGGCAATGCTG	*HXL1* sequencing primer 1 (*C. deuterogattii*)
B5806	GCAAAGAAGCCAGAGTGAG	*HXL1* sequencing primer 2 (*C. deuterogattii*)
B5807	CGTCTTCCCAAAATGTATCG	*HXL1* sequencing primer 3 (*C. gattii*)
B5251	CACTCCATTCCTTTCTGC	RT-PCR primer L1 for *HXL1* of *C. neoformans*
B5252	CGTAACTCCACTGTGTCC	RT-PCR primer R1 for *HXL1* of *C. neoformans*
B5757	CGTAACTCTACTGTCTCC	RT-PCR primer L1 for *HXL1* of *C. deneoformans*
B5758	ATTTCCTCCCTTCCAAGC	RT-PCR primer R1 for *HXL1* of *C. deneoformans*
B5759	CACTCCATCCCCTTCAGC	RT-PCR primer L1 for *HXL1* of *C. deuterogattii*
B5760	CGTAACTCTATCGTCTCG	RT-PCR primer R1 for *HXL1* of *C. deuterogattii*
C51	CTTCCAGCCTTCTCTCCTTG	RT-PCR primer L1 for *ACT1*
C52	AGAGGTCCTTCCTGATGTCG	RT-PCR primer R1 for *ACT1*
B677	AATCTCCTTACCAGCCATTCGG	qRT primer 1 for *ERG11* of *C. neoformans*
B678	TTCAGGGAACTTGGGAACAGC	qRT primer 2 for *ERG11* of *C. neoformans*
B6875	AATGGTCAGTTCGCCAAG	qRT primer 1 for *ERG11* of *C. deneoformans*
B6332	CTCATCTCGCCTTTTGTAAC	qRT primer 2 for *ERG11* of *C. deneoformans*
B6877	ATGCCAAGGTCTGGAATC	qRT primer 1 for *ERG11* of *C. deuterogattii*
B6334	AGAACCGAAACCATAGTCG	qRT primer 2 for *ERG11* of *C. deuterogattii*
B1720	ATCCCTTTTCACCGTCGCTC	qRT primer 1 for *ERG3* of *C. neoformans*
B6839	GTGATGTTCTAATCTTCTA	qRT primer 2 for *ERG3* of *C. neoformans*

J595	GGCGTTGAGCGTGTTTATG	qRT primer 1 for *ERG3* of *C. deneoformans*
J596	GGCTTGTGGATGTGCTTG	qRT primer 2 for *ERG3* of *C. deneoformans*
J593	GTAGATGGTTACGCCCAATCC	qRT primer 1 for *ERG3* of *C. deuterogattii*
J594	CAGAACTGGACAAAGACGAAG	qRT primer 2 for *ERG3* of *C. deuterogattii*
C53	CTCTGAGGACGACAAGGACA	qRT primer 1 for *KAR2* of *C. neoformans*
C54	AGCTCAGAAAGCTGCTCCTC	qRT primer 2 for *KAR2* of *C. neoformans*
J444	CGAGGAGCAGCTTTCTGAG	qRT primer 1 for *KAR2* of *C. deneoformans*
B4275	TCATGGCTGAAAGGCATC	qRT primer 2 for *KAR2* of *C. deneoformans*
J445	TTCTGAGGACGACAAGGACA	qRT primer 1 for *KAR2* of *C. deuterogattii*
B5254	AGCTCAGAAAGCTGCTCCTC	qRT primer 2 for *KAR2* of *C. deuterogattii*
B679	CGCCCTTGCTCCTTCTTCTATG	qRT primer 1 for *ACT1* of *C. neoformans*
B680	GACTCGTCGTATTCGCTCTTCG	qRT primer 2 for *ACT1* of *C. neoformans*
B6411	GACAATGGCTCTGGTATGTG	qRT primer 1 for *ACT1* of *C. deneoformans*
B6876	CAACAATAGAAGGGAAGACAGC	qRT primer 2 for *ACT1* of *C. deneoformans*
B6878	CGGTATCGTCACAAACTGG	qRT primer 1 for *ACT1* of *C. deuterogattii*
B6879	TAAGAAGAACGGGGTGCTC	qRT primer 2 for *ACT1* of *C. deuterogattii*
J792	TGCTCGTGTCCTCTGTTG	qRT primer 1 for *DER1* of *C. deuterogattii*
J793	AGAGACATCTTCACGGAAGG	qRT primer 2 for *DER1* of *C. deuterogattii*
J794	CGCCTCCAACAAAGACAAG	qRT primer 1 for *ALG7* of *C. deuterogattii*
J795	TGATGTGAACCTGTTGGTCC	qRT primer 2 for *ALG7* of *C. deuterogattii*
J796	CCGTAGTTCAGATGGAAGAGTC	qRT primer 1 for *PMT1* of *C. deuterogattii*
J797	CGGATCACCTGCATCGTTC	qRT primer 2 for *PMT1* of *C. deuterogattii*
J800	CACTACCTTCCCGCTCATC	qRT primer 1 for *PMT4* of *C. deuterogattii*
J801	CGGACCGGCAATACTGATG	qRT primer 2 for *PMT4* of *C. deuterogattii*
J802	TTCCACGCCGATGACATCC	qRT primer 1 for *WBP1* of *C. deuterogattii*
J803	ATGTTGTGGCATCGGCAG	qRT primer 2 for *WBP1* of *C. deuterogattii*
J804	CCTCGATACCCTCTGTTGG	qRT primer 1 for *OST1* of *C. deuterogattii*
J805	CGTTCTTCCCATTGGCCTG	qRT primer 2 for *OST1* of *C. deuterogattii*
J806	TATGACTCTTGCAGGGCAC	qRT primer 1 for *SEC61* of *C. deuterogattii*
J807	CTCAACAAGCCAAGTGTCG	qRT primer 2 for *SEC61* of *C. deuterogattii*
J808	CGGCTTCAAGGCCAAATG	qRT primer 1 for *ERV29* of *C. deuterogattii*
J809	CTGAGGGTGAGCAGAATGG	qRT primer 2 for *ERV29* of *C. deuterogattii*
J810	CACCACTCCCAATCAGGAC	qRT primer 1 for *SOD2* of *C. deuterogattii*
J811	TCGGGCTTGACGTTCTTG	qRT primer 2 for *SOD2* of *C. deuterogattii*
J812	ATGGCAATGATCCGCTTCG	qRT primer 1 for *PPS1* of *C. deuterogattii*
J813	TCCCACACGACAATGAACC	qRT primer 2 for *PPS1* of *C. deuterogattii*
J814	GGAAAGGAAATGGCCGAAG	qRT primer 1 for *CHS2* of *C. deuterogattii*
J815	TTCTCTTTCACAGGCACCATC	qRT primer 2 for *CHS2* of *C. deuterogattii*

References

1 Perfect, J. R., Ketabchi, N., Cox, G. M., Ingram, C. W. & Beiser, C. L. Karyotyping of *Cryptococcus neoformans* as an epidemiological tool. *J. Clin. Microbiol.* **31**, 3305-3309 (1993).

2 Cheon, S. A. *et al.* Unique evolution of the UPR pathway with a novel bZIP transcription factor, Hxl1, for controlling pathogenicity of *Cryptococcus neoformans*. *PLoS Pathog.* **7**, e1002177, doi:10.1371/journal.ppat.1002177 (2011).

3 Moore, T. D. & Edman, J. C. The alpha-mating type locus of *Cryptococcus neoformans* contains a peptide pheromone gene. *Mol. Cell. Biol.* **13**, 1962-1970 (1993).

4 Jung, K. W., So, Y. S. & Bahn, Y. S. Unique roles of the unfolded protein response pathway in fungal development and differentiation. *Sci. Rep.* **6**, 33413, doi:10.1038/srep33413 (2016).

5 Kidd, S. E. *et al.* A rare genotype of *Cryptococcus gattii* caused the cryptococcosis outbreak on Vancouver Island (British Columbia, Canada). *Proc. Natl. Acad. Sci. USA* **101**, 17258-17263, doi:10.1073/pnas.0402981101 (2004).
